# Enhancing face validity of mouse models of Alzheimer’s disease with natural genetic variation

**DOI:** 10.1371/journal.pgen.1008155

**Published:** 2019-05-31

**Authors:** Kristen D. Onos, Asli Uyar, Kelly J. Keezer, Harriet M. Jackson, Christoph Preuss, Casey J. Acklin, Rita O’Rourke, Rebecca Buchanan, Travis L. Cossette, Stacey J. Sukoff Rizzo, Ileana Soto, Gregory W. Carter, Gareth R. Howell

**Affiliations:** 1 The Jackson Laboratory, Bar Harbor, Maine, United States of America; 2 Department of Biomedical and Translational Sciences, Rowan University, Glassboro, New Jersey, United States of America; 3 Sackler School of Graduate Biomedical Sciences, Tufts University School of Medicine, Boston, Massachusetts, United States of America; 4 Graduate School of Biomedical Sciences and Engineering, University of Maine, Orono, Maine, United States of America; Columbia University Medical Center, UNITED STATES

## Abstract

Classical laboratory strains show limited genetic diversity and do not harness natural genetic variation. Mouse models relevant to Alzheimer’s disease (AD) have largely been developed using these classical laboratory strains, such as C57BL/6J (B6), and this has likely contributed to the failure of translation of findings from mice to the clinic. Therefore, here we test the potential for natural genetic variation to enhance the translatability of AD mouse models. Two widely used AD-relevant transgenes, *APP*^*swe*^ and *PS1*^*de9*^ (*APP/PS1*), were backcrossed from B6 to three wild-derived strains CAST/EiJ, WSB/EiJ, PWK/PhJ, representative of three *Mus musculus* subspecies. These new AD strains were characterized using metabolic, functional, neuropathological and transcriptional assays. Strain-, sex- and genotype-specific differences were observed in cognitive ability, neurodegeneration, plaque load, cerebrovascular health and cerebral amyloid angiopathy. Analyses of brain transcriptional data showed strain was the greatest driver of variation. We identified significant variation in myeloid cell numbers in wild type mice of different strains as well as significant differences in plaque-associated myeloid responses in *APP/PS1* mice between the strains. Collectively, these data support the use of wild-derived strains to better model the complexity of human AD.

## Author summary

Despite the rise in incidence of Alzheimer’s disease (AD), it has been over a decade since a new drug treatment has been introduced. Recently, a number of pharmaceutical giants have shut down their AD research units. One issue that these companies and researchers have struggled with is the lack of translatability of preclinical studies to the clinic. One aspect that has come under heavy scrutiny is whether the mouse can be an appropriate model for a complex human disease such as AD. Current mouse models of AD have incorporated well-known early onset AD mutations on a single genetic background, C57BL/6J, which does not develop all features of human AD- namely marked neurodegeneration. Here we sought to improve the utility and translatability of mouse models through the use of three genetically distinct, wild-derived inbred mouse strains, CAST/EiJ, WSB/EiJ and PWK/PhJ. These mice encompass millions of genetic differences that have never before been explored in the context of modeling AD. Wild-derived mice that carried the early onset AD mutations exhibited robust differences in immune response to amyloid, evidence of mixed pathology and early neurodegeneration, better recapitulating what happens in human AD than previous models.

## Introduction

Alzheimer’s disease (AD) is the most common cause of adult dementia, with approximately 6 million Americans diagnosed with either clinical AD or mild cognitive impairment in 2017[1]. Age is the greatest risk factor and currently we have the largest aging population that has ever been on this planet [2]. Globally, there are 50 million people living with dementia and this number is expected to reach 152 million by 2050. Low- and middle-income countries are the hardest hit, comprising 66% of global cases [3]. AD is pathologically characterized by the accumulation of beta amyloid (β-amyloid) plaques, neurofibrillary tangles, and widespread neuronal loss. Another prominent feature is the neuroinflammatory response by a variety of cells including astrocytes and microglia. Multiple studies have identified two forms of AD: familial AD (FAD, also known as early-onset AD) and sporadic AD (also known as late-onset AD). Widely-used mouse models of AD utilize FAD mutations in amyloid precursor protein (*APP*) and presenilin 1 and 2 (*PSEN1* and *PSEN2*). However, a recent review on the current status of AD clinical trials has suggested that the failure of these trials, in part, is due to the inability of current AD mouse models to translate to humans [4]. While FAD mouse models have been vital to understand early drivers of amyloidosis, to date, they do not effectively model all hallmarks of AD, particularly frank neurodegeneration. This has led some to question the utility of mouse models as preclinical models for AD and other diseases of complex etiologies.

Studies including the Dominantly Inherited Alzheimer’s Network (DIAN) and the Religious Order Study and Memory and Aging Project (ROSMAP) show significant variation in age of onset and rate of disease progression in individuals who inherit the same FAD mutations[5, 6]. Furthermore, new work performing a genome-wide association study (GWAS) [7] on individuals with a family history of AD identified multiple novel variants. This suggests that the underlying genetic contribution of many cases of FAD are also due to multiple interacting variants, not simply the single strong variants such as those in *APP*, *PSEN1* and *PSEN2*. Therefore, the same is likely true in mouse models.

Murine models relevant to AD have been almost exclusively developed on a single genetic background, C57BL/6 (B6). Few studies have assessed FAD mutations in a limited number of alternative genetic backgrounds including 129S1/SvImJ [8], A/J and DBA/2J (D2) [8–10]. These studies showed genetic background altered β-amyloid deposition and seizure incidence, but modifications to neuronal cell loss were not reported. Supporting the potential of incorporating genetic variation in AD mouse models, a recent study used F1 crosses between B6 and thirty classical inbred strains to show that the phenotypes observed from a heterozygous null mutation related to neurological function were not generalizable across strain [11]. Interestingly, there were multiple cases in which there were inverse effects of the same allele on phenotypic outcomes. Another recent publication showed greater transability of the mouse to human Alzheimer’s through the development of a new mouse panel known as the AD-BXDs. This panel was developed by crossing congenic B6 5xFAD mice with BxD males (B6xD2), greatly increasing the genetic diversity in the context of 5 aggressive familial mutations. Aging and characterization of these mice indicated a greater range in AD related phenotypes such as plaque pathology and cognitive deficits, and a greater transcriptional overlap with human AD [12]. These studies highlight the likely huge potential for generating more translatable AD mouse models through the use of different genetic contexts.

**Fig 1:**
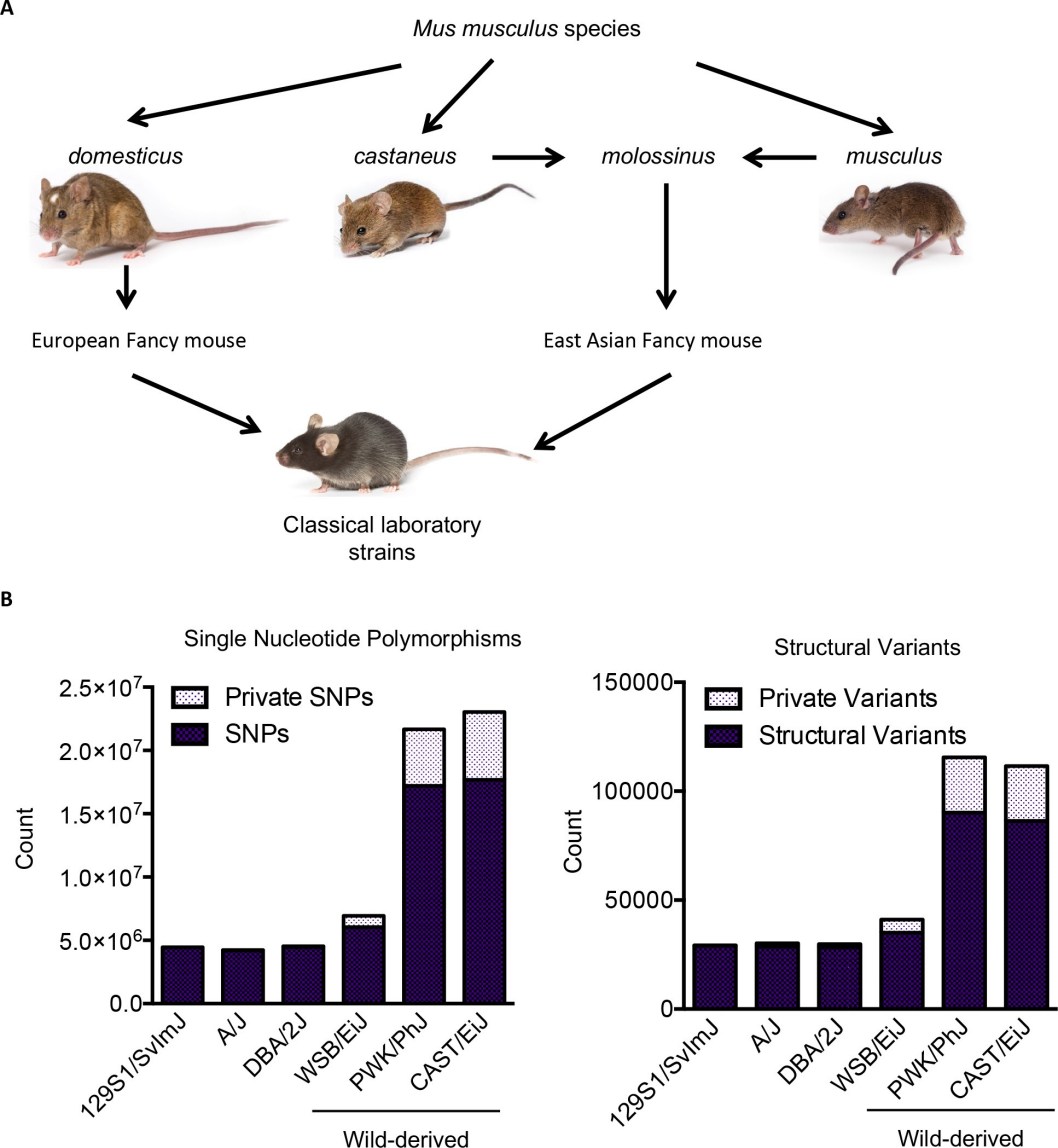
Wild-derived mice are genetically distinct. (A) Wild-derived mouse strains are genetically distinct from classical laboratory mouse strains and are representative of the three main *Mus musculus* subspecies *musculus*, *castaneus*, and *domesticus* that diverged from a common ancestor about one million years ago [62]. Classical laboratory strains were developed over 100 years ago from crosses between domesticated European fancy mice and East Asian fancy mice[63]. Wild-derived inbred strains were caught in the wild and inbred less than 50 years ago. Mouse photos are used with permission from The Jackson Laboratory. (B) The number of single nucleotide polymorphisms (SNPs) and structural variants in reference to C57BL/6J in three classical laboratory strains previously used to assess Alzheimer’s disease mutations [8–10], and the three wild-derived strains used in this study. Private SNPs and variants refer to those that are only found in that individual strain. Data is derived from [14].

Therefore, to take full advantage of the level of natural genetic variation available in mice, we employed genetically distinct wild-derived strains. Historically, the lineage of commonly used classical laboratory strains can be traced to domesticated fancy mouse stock developed on a farm in Massachusetts in the early 1900s [13]. Due to this, classical laboratory strains are undefined genomic mixtures of two or more subspecies of *Mus musculus* (including *Mus musculus domesticus and Mus musculus molossinus*). They exhibit limited inter-strain polymorphisms (less than 5 million differences between a classical inbred strain when compared to B6/J ([14] and **Fig 1**), and do not represent any animal that exists in nature. To overcome the limitations of classic laboratory strains, ‘wild-derived’ strains were introduced as research models in the 1980s. Wild-derived strains are genetically distinct subspecies of *Mus musculus* (e.g. *Mus musculus musculus* and *Mus musculus castaneous*). Founders of each strain were caught from well-established wild mice populations from around the world (see methods), and then inbred [15]. Wild-derived strains show a much greater degree of genetic variation compared to B6 than other classical inbred strains do (between 6 and 17 million differences) including millions of private variations. Importantly, the genetic variation encompassed in these strains and interactions of different gene networks evolved, thus, are likely physiologically relevant to the natural world. This variation includes genes previously associated with AD including *Apoe*, *Trem2* and *Tyrobp*, and these strains also show variation in phenotypes relevant to AD risk factors including cardiovascular health [16], insulin secretion [17–19], gut microbiota [19, 20] and circadian rhythm [21].

In this study, we hypothesized that incorporating FAD mutations into genetically distinct, wild-derived mouse strains would establish more clinically-relevant AD mouse models compared to those on classic laboratory strain backgrounds. To test this, two commonly used FAD mutations (*APP*^*swe*^ and *PSEN1*^*de9*^, herein referred to as *APP/PS1*) were introduced into three wild-derived strains representative of the three *Mus musculus (mus)* subspecies: WSB/EiJ (WSB, *M*. *mus domesticus*), PWK/PhJ (PWK, *M*. *mus musculus*), and CAST/EiJ (CAST, *M*. *mus castaneus*). Assessment of AD-relevant phenotypes showed that the effects of the *APP/PS1* transgenes are strain-dependent and sex-dependent, with significant differences in amyloid deposition, neuronal cell loss and cerebral amyloid angiopathy (CAA). Transcriptional profiling and neuropathological assessment suggested myeloid cell responses are major contributors to the variation in AD phenotypes we observed in the wild-derived AD strains.

## Results

### Strain- and sex- and genotype-specific differences in physiology and functional outcomes in wild-derived AD models

Three wild-derived AD mouse models were created by backcrossing for at least six generations the *APP/PS1* transgenes from B6 to the genetically distinct substrains WSB, PWK and CAST (Fig 1). The presence of both the *APP*^*swe*^ and *PSEN1*^*de9*^ transgenes was confirmed by PCR (S1 Fig). For each strain, balanced cohorts of female and male wild type (WT) and *APP/PS1* mice were established and aged to 6 months–an age window when the majority of plaques have seeded and are in an exponential growth phase in B6.*APP/PS1* [22–24]. *APP/PS1* and randomized WT litter mate controls from each strain were tested sequentially in the following order: (1) PWK, (2) WSB, (3) CAST and (4) B6. For this first characterization of these new strains, a set of metabolic and functional assays were selected that spanned across a wide-range of AD-relevant phenotypes. Significant strain-, sex- or genotype-specific differences were observed in body weight, body temperature and body composition (S2 Fig). In addition, significant differences were observed in activity measured using both piezoelectric floor monitoring and open field arenas (S3 Fig). WT mice from all three wild-derived strains were significantly more active than B6 WT mice. Also, irrespective of genetic context, all *APP/PS1* strains showed the previously reported increase in activity [25] compared to their WT counterparts.

Cognitive function was assessed in wild-derived strains and B6 using spontaneous alternation (working memory) and novel spatial recognition (short-term memory) in a Y-maze. Given the increased behavioral ‘wildness’ [26, 27] of the wild-derived strains, the Y-maze was modified to include specially fabricated covers (see Methods) to minimize likelihood of escape. This was the first time that these tasks had been employed by us for either aging B6 mice or wild-derived strains of any age. However, these tasks had been previously validated using young B6 male mice [28] and further validated here using PWK (the first wild-derived strain to be tested). For spontaneous alternation (S4A–S4C Fig), percent alternation exceeded 50% for all strains irrespective of genotype. Despite hyperactivity phenotypes observed in open field in wild-derived mice, there were no transgenic-related differences in activity levels as measured by total arm entries, thus, increased activity does not confound the interpretation of this task. Furthermore, we found no correlation between number of arm entries and performance. Therefore, these data suggest working memory was not affected by the *APP/PS1* transgenes. For novel spatial recognition (S4D–S4F Fig), strain-, sex- and genotype-specific differences were observed. For the PWK strain, a robust preference for the novel arm after a 30-minute delay was shown for both male and female WT and *APP/PS1* mice indicating an intact short-term memory. In contrast, for WSB females and CAST males, WT but not *APP/PS1* mice showed a preference for the novel arm suggesting working memory was impaired in both female WSB.*APP/PS1* and male CAST.*APP/PS1* mice. Highlighting the challenges of identifying tasks that can be performed by diverse strains, short-term memory using this task could not be determined for male WSB.*APP/PS1*, female CAST.*APP/PS1*, and male and female B6.*APP/PS1*, as the strain-matched and sex-matched WT counterparts were unable to perform the task.

### Neuronal cell loss observed in WSB and CAST AD models

**Fig 2:**
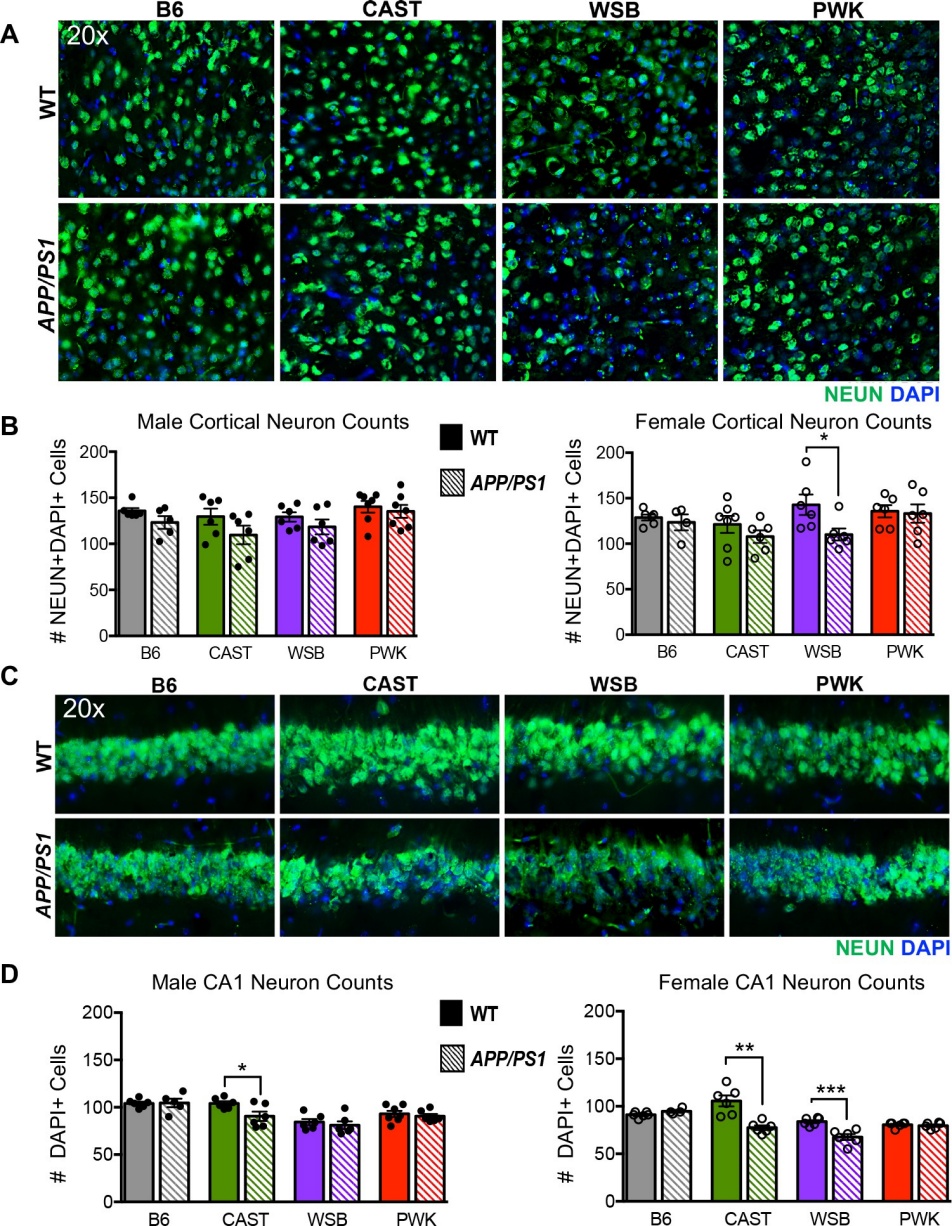
Cortical and Hippocampal neuronal loss in wild-derived *APP/PS1* mice. (A) Representative 20x images of NEUN+DAPI+ staining in superior cortex utilized for cell counts. (B) There were significantly fewer neurons in the cortex of female WSB.*APP/PS1* compared to female WSB WT mice (p ≤ 0.05). No neuronal cell loss was observed in the cortex of either female B6.*APP/PS1*, CAST.APP/PS1, PWK.APP/PS1 mice or male mice of any strain. See S1 Table for associated statistical analyses in supporting information. (C) Representative 20x images of NEUN+DAPI+ staining of the CA1 pyramidal layer of hippocampus utilized for cell counting. (D) Male and female CAST.*APP/PS1* (male p ≤ 0.05; female p ≤ 0.01) and female WSB.*APP/PS1* (p ≤ 0.001) mice exhibited significant neuronal cell loss in the CA1 region of the hippocampus. There was no neuronal cell loss in the CA1 region of the hippocampus of both B6.*APP/PS1* and PWK.*APP/PS1*. See S1 Table for associated statistical analyses in supporting information.

Next, to assess neurodegeneration, NEUN+DAPI+ cell counts were performed across all strains, sexes and genotypes in a region of the superior cortex and in the CA1 region of the hippocampus, two brain regions commonly affected early in human AD (**Fig 2, S1 Table**). Interestingly, even in the absence of the *APP/PS1* transgenes, strain background was a significant driver of the overall neuronal cell number in the CA1 region. Importantly, there was a significant loss of NEUN+DAPI+ cells in female WSB.*APP/PS1* in the cortical region and CA1 compared to WT WSB females. There was also significant loss of neurons in male and female CAST.*APP/PS1* mice in the CA1 region. There was no detectable NEUN+DAPI+ loss in either B6.*APP/PS1* (as previously published in [10, 29, 30]) or PWK.*APP/PS1* strains in the two regions studied. Despite the presence of neurodegeneration in CAST.*APP/PS1* and female WSB.*APP/PS1*, there was no evidence of increased tau pathology using AT8, a marker of early tau hyperphosphyloration (S5 Fig).

### Evidence of mixed pathologies in wild-derived *APP/PS1* strains

**Fig 3:**
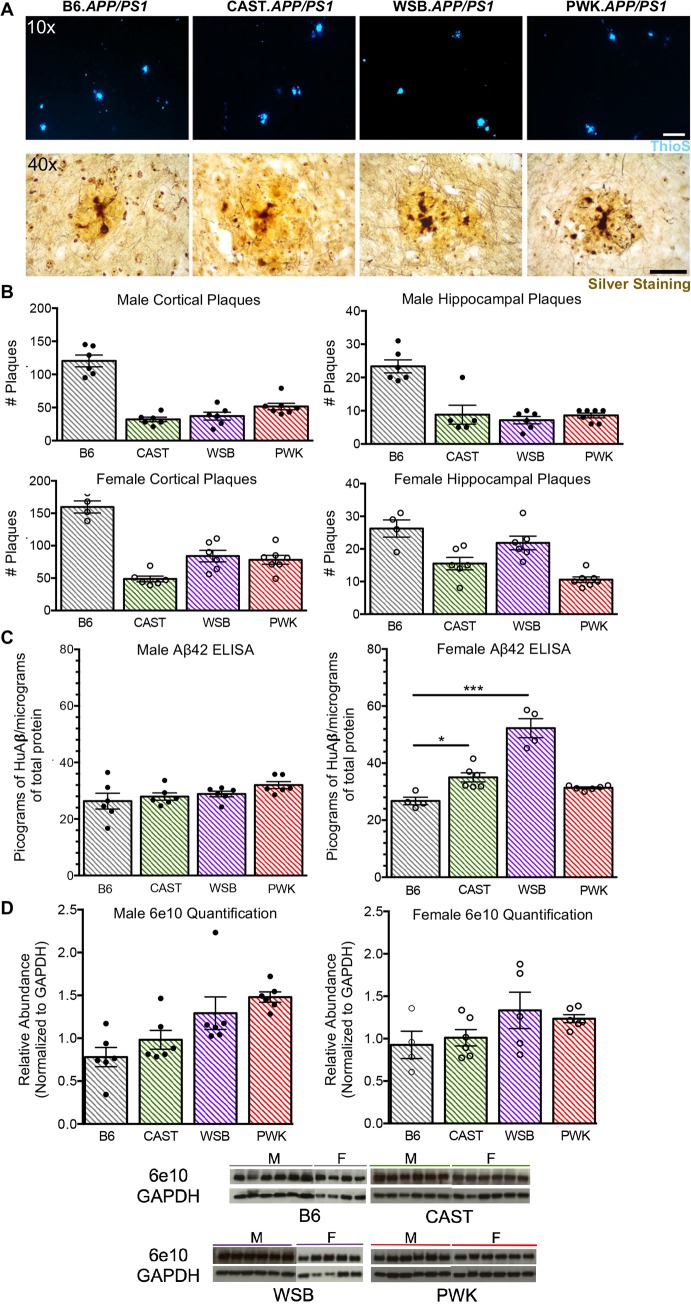
Differences in plaque counts and amyloid deposition. (A) Representative 10x images of ThioS+ cortical plaques with scale bar representing 100 microns. Representative 40x bright field images of plaques with silver staining. There was no evidence of mature neurofibrillary tangles of tau in any of the *APP/PS1* strains. (B) Cortical and hippocampal plaque counts were performed on *APP/PS1* strains. There was no evidence of ThioS+ staining in WT animals. The number of plaques were compared in male and female *APP/PS1* mice across strains. For cortical counts, female *APP/PS1* mice of any background strain exhibited significantly more plaques than male counterparts (B6.*APP/PS1* p ≤ 0.05; CAST.*APP/PS1* p ≤ 0.05; WSB.*APP/PS1* p ≤ 0.01; PWK.*APP/PS1* p ≤ 0.01). For hippocampal plaque counts, only WSB.*APP/PS1* mice showed significantly higher plaque counts than male counterparts (p ≤ 0.0001). See S2 Table for associated statistical analyses in supporting information. (C) Results from Aβ42 ELISA revealed that female CAST.*APP/PS1* and WSB.*APP/PS1* had significantly higher picograms (pg) of human Aβ42 per milligram (mg) of total protein than B6.*APP/PS1* (CAST.*APP/PS1* p ≤ 0.05; WSB.*APP/PS1* p ≤ 0.001). (D) Western Blots of 6e10 (100 kDa) and GAPDH (36 kDa) across all strains. Male PWK.*APP/PS1* exhibited significantly higher APP protein than male B6.*APP/PS1* (p ≤ 0.01). All other transgenic animals were not different.

Amyloidosis was assessed in all four strains using ThioS staining, ELISA and Western blotting. Surprisingly, numbers of cortical ThioS+ plaques were significantly decreased in all three of the wild-derived *APP/PS1* strains in comparison with B6.*APP/PS1* (**Fig 3A–3C**, **S2 Table)**. Numbers of hippocampal ThioS+ plaques were also significantly decreased with the exception of WSB.*APP/PS1* females. No plaques were observed in WT mice from any of the four strains in any brain region. Plaque morphology appeared different between B6.*APP/PS1* and wild-derived *APP/PS1* strains. Specifically, there was an absence of small ThioS+ plaques in wild-derived *APP/PS1* compared to B6.*APP/PS1* mice. Despite the reduced numbers of plaques, there was a significant increase in Aβ42 (measured by ELISA) in both female CAST.*APP/PS1* and WSB.*APP/PS1* compared to B6.*APP/PS1* (**Fig 3D**). This increase cannot be accounted for simply by differences in mutant APP production as Western blotting using 6e10 (antibody to human mutant APP) showed similar APP protein levels across all strains (**Fig 3E**) with the exception of male PWK.*APP/PS1* (significant difference between male B6.*APP/PS1* and male PWK.*APP/PS1*, p ≤ 0.01). Therefore, our data suggest that at 8 months, plaques in the wild-derived *APP/PS1* strains may be further along in the rapid growth period previously defined for B6.*APP/PS1* mice [24].

**Fig 4:**
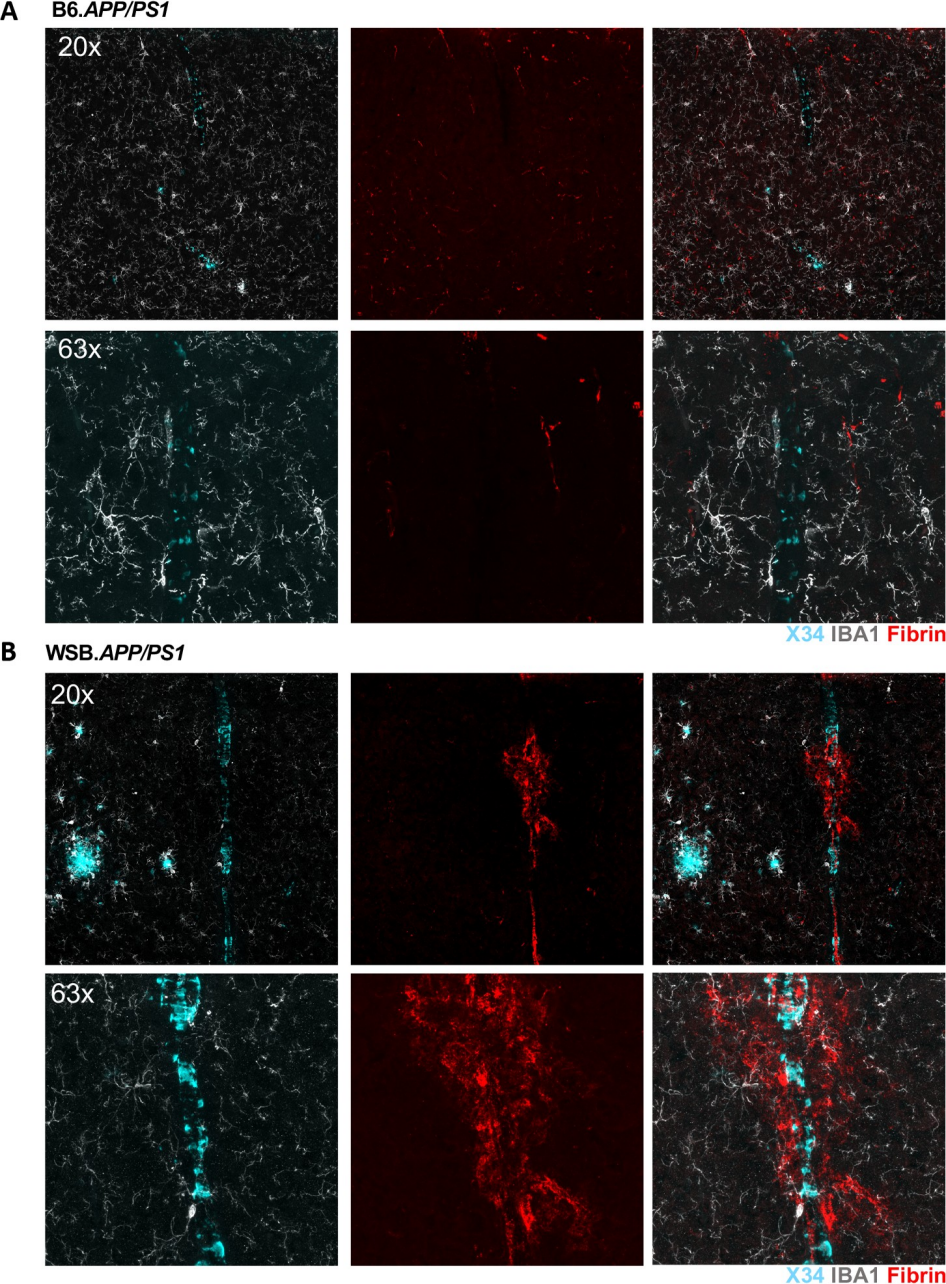
Cerebral amyloid angiopathy and decreased cerebrovascular integrity in WSB.*APP/PS1*. (A) Limited CAA in a female 8-month B6.*APP/PS1*. While light X34 staining was present in some vessels, complete banding characteristic of CAA has not observed in any vessels. Fibrin staining was restricted to the inside of vessels suggesting the blood brain barrier remained intact in B6.*APP/PS1* mice. (B) Severe CAA in a female 8-month WSB.*APP/PS1*. X34 staining demonstrated thick banding throughout the majority of vessels. Fibrin staining outside of the blood vessels suggested decreased cerebrovascular integrity in WSB.*APP/PS1* mice.

Another prominent amyloid phenotype observed in the wild-derived strains was ThioS+ vessels, suggesting the occurrence of cerebral amyloid angiopathy (CAA). Brain sections from all strains, sexes and genotypes were examined for the presence of ThioS+ vessels and by silver staining. CAA was pronounced in vessels of CAST.*APP/PS1* and WSB.*APP/PS1*, but not B6.*APP/PS1* or PWK.*APP/PS1* mice (S6 Fig). There was no evidence of vascular staining of ThioS in WT animals. CAA has been associated with cerebrovascular damage in human AD and recent studies support a more prominent role of cerebrovascular decline in AD pathogenesis [31, 32]. To test the relationship between CAA and cerebrovascular integrity, brain sections from WSB.*APP/PS1* were assessed as they showed the greatest percentage of ThioS+ vessels. Cerebrovascular integrity was determined using antibodies to fibrin(ogen), a protein that is ordinarily present in blood but its presence in the brain is indicative of blood brain barrier compromise. Fibrin was present outside of the microvessels in brain sections from WBS.*APP/PS1*, but not in B6.*APP/PS1* (**Fig 4).**

### Transcriptional profiling identifies significant strain-specific neuroinflammatory signatures

**Fig 5:**
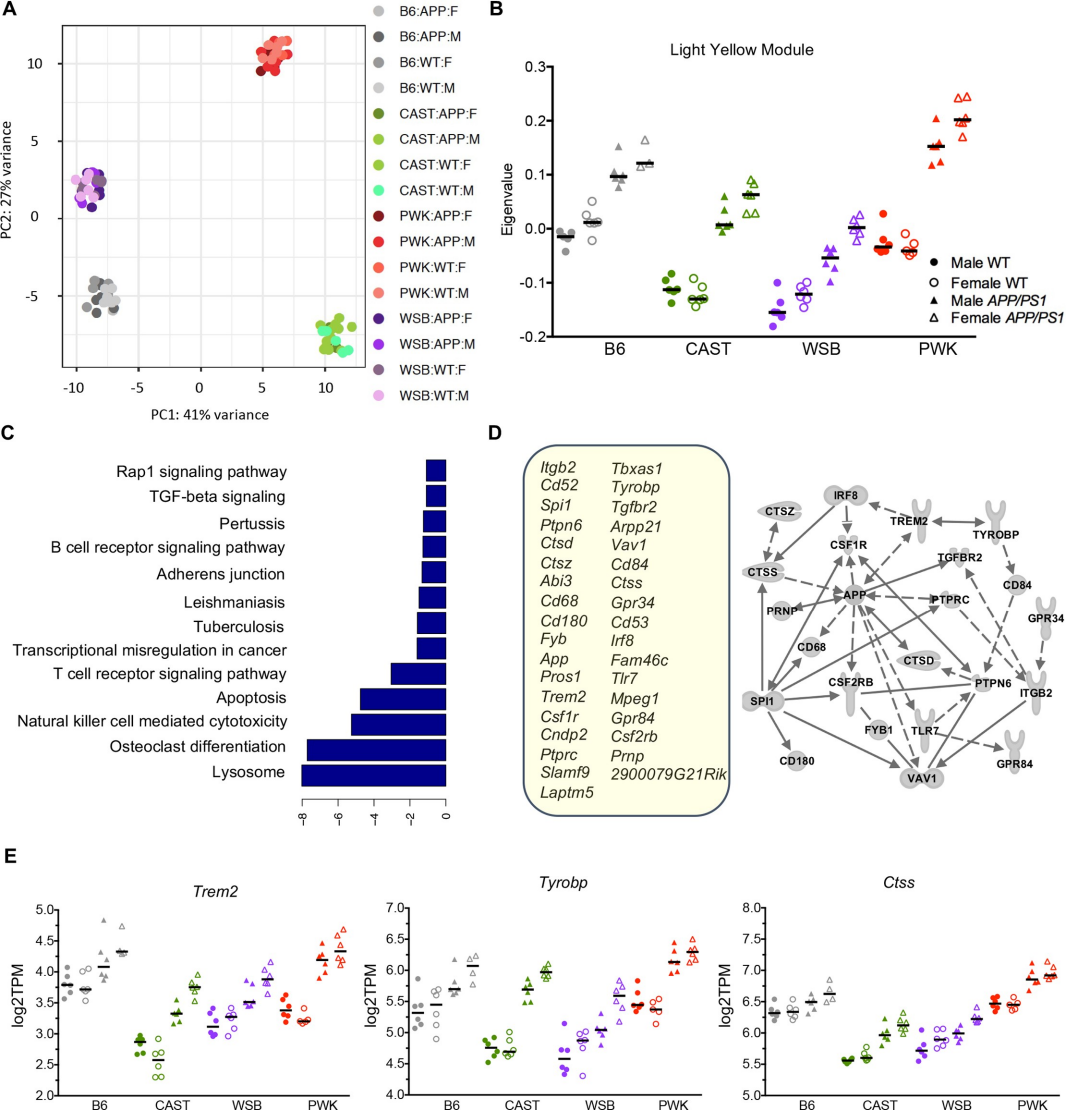
Transcriptional profiling identifies strain differences in myeloid gene expression. (A) Principle component analysis identifies strain as the greatest driver of gene expression variation. (B) Weighted Gene Co-expression Analysis (WGCNA) identified one transgene specific ‘Light Yellow’ module across the mouse panel. Eigenvalues suggest variation in expression in WT strains, and magnitude difference in expression comparing transgenic to WT samples. (C) Genes contained within the ‘Light Yellow’ module are enriched for Lysosome and Osteoclast Differentiation KEGG pathways. (D) The list of 35 genes within the module contained many myeloid related genes previously associated with human AD. Ingenuity Pathway Analyses indicates that these identified genes all fit within the APP network. (E) Plots of gene expression levels to demonstrate variation across the strains. In WT samples, *Trem2*, *Tyrobp* and *Ctss* showed the lowest expression in WSB and CAST, two strains that showed amyloid-induced neuronal cell loss. The magnitude of expression difference between WT and *APP/PS1* mice also varies between strains.

To provide insight into the strain-specific differences that may be driving the phenotype differences observed between strains, transcriptional profiling by RNA-seq was performed on the left brain hemispheres from WT and *APP/PS1* male and female mice from all strains (93 samples in total). Sequencing depth (S7 Fig) and expression levels of the *APP*^*swe*^ and *PSEN1*^*de9*^ transcripts in *APP/PS1* mice (S8 Fig) were consistent between strains. Principle Component Analysis (PCA) identified strain as the greatest driver of gene expression variance across samples, consistent with the genetic distinctness of strains (**Fig 5A**). To identify modules of genes that were differentially expressed between groups, Weighted Gene Co-expression Analysis (WGCNA) was performed. The majority of modules were driven by strain, independent of *APP/PS1* genotype (S9 Fig). However, one module (termed ‘light yellow’) was driven by *APP/PS1* genotype and seen in all strain backgrounds (**Fig 5B**). This module contained 35 genes that are enriched for the Lysosome and Osteoclast Differentiation KEGG pathways (**Fig 5C**). The light yellow module included *App* and *Psen1* supporting the fact that this module is likely an amyloid response module. The majority of other genes in the module are expressed in myeloid cells (either resident microglia and/or monocytes/macrophages). Many of these genes have been previously implicated in AD-relevant processes such as amyloid deposition and synaptic loss including *C1qa*, *Csf1r*, *Tyrobp*, *Cx3cr1*, *Cd68* and *Ctsz*. Importantly, DNA variations in two genes in the light yellow module, *Trem2* and *Cd33*, have previously been associated with human AD suggesting these genes may be early drivers of AD pathogenesis.

Assessment of the eigenvalues for the light yellow module revealed two major findings. First, there was great variation in the eigenvalues when comparing WT samples between strains. For instance, eigenvalues were lowest for WT samples from WSB and CAST. This was reflected in the normalized expression levels of genes in the module. *Trem2*, *Tyrobp* and *Ctss* showed the lowest expression in WT samples from WSB and CAST (**Fig 5E**)–strains that showed neuronal cell loss in the presence of amyloid (**Fig 2**). The second major finding was that there were marked differences in eigenvalues comparing WT to *APP/PS1* samples. The greatest difference between WT and *APP/PS1* samples was observed in PWK. Again, these differences were also observed at the level of individual genes within the light yellow module (**Fig 5B**). This suggests the ability of myeloid cells to respond to amyloid is strongly influenced by genetic context. Together, these data suggest that there are intrinsic differences between myeloid cells in WT samples from different strains and that these cells respond differently to amyloid deposition. Both these factors are likely critical in determining whether or not a strain is susceptible to amyloid-induced neurodegeneration.

### Genetic context modifies myeloid cells in WT and *APP/PS1* mice

**Fig 6:**
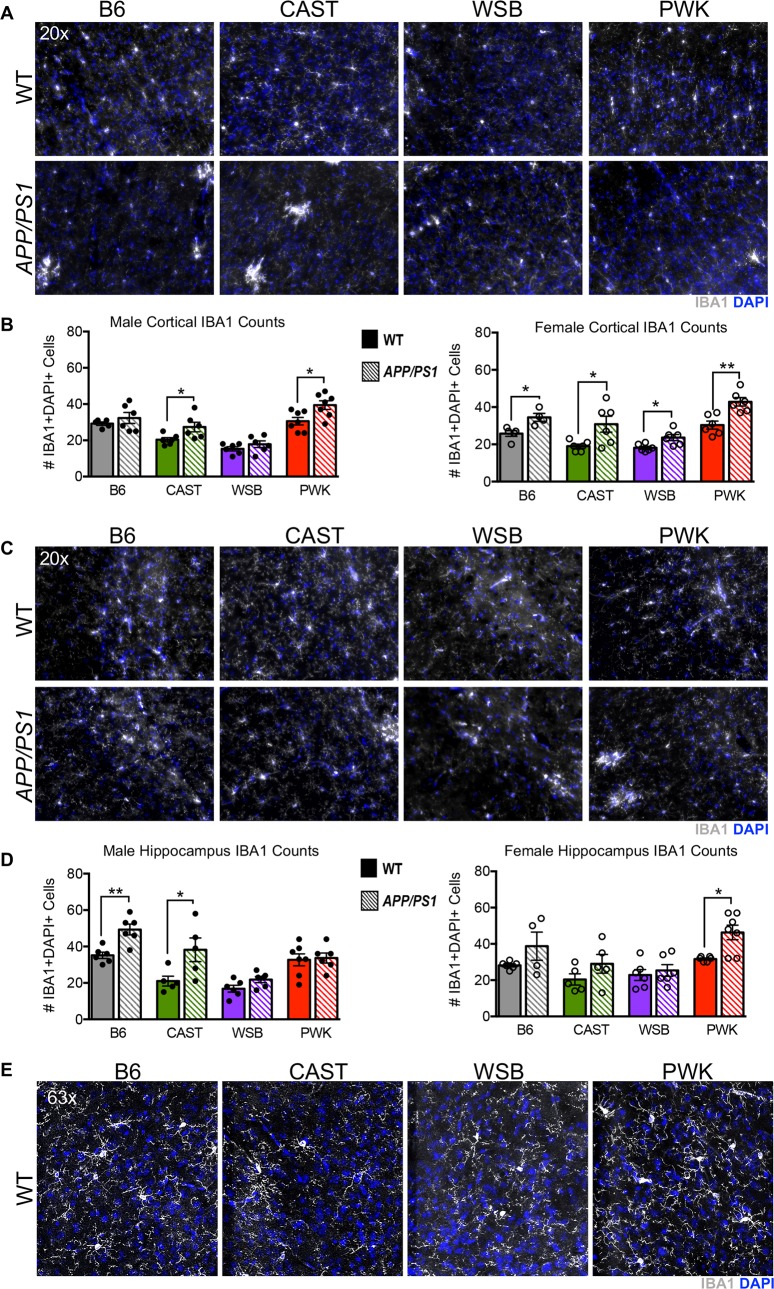
Myeloid cell numbers are different across strains. (A) Representative 20x images of IBA1+DAPI+ staining in superior cortex utilized for cell counts. (B) In the cortex, there was a significant increase in IBA1+ cells in male CAST.*APP/PS1* and PWK.*APP/PS1* in comparison to WT counterparts (p ≤ 0.05). There was a significant increase in IBA1+ cells in female *APP/PS1* mice for all strains in comparison to WT counterparts (*APP/PS1* for B6, CAST and WSB: p ≤ 0.05; PWK.APP/PS1: p ≤ 0.01). See S3 Table for associated statistical analyses in supporting information. (C) Representative 20x images of IBA1+DAPI+ staining in hippocampus utilized for cell counts. (D) In the hippocampus, there was a significant increase in IBA1+ cells in male B6.*APP/PS1* (p ≤ 0.01) and CAST.*APP/PS1* (p ≤ 0.05) in comparison to WT counterparts. There was a significant increase in IBA1+ cells in female PWK.*APP/PS1* mice in comparison to WT counterparts (p ≤ 0.05). See S3 Table for associated statistical analyses in supporting information. (E) Representative 63x images of IBA1+DAPI+ cortical staining in WT animals.

A major and unexpected finding from the transcriptional profiling was that transcript levels of myeloid cell genes were significantly lower in WT WSB and CAST mice compared to B6 and PWK mice. This suggests that myeloid cells vary between strains, even in the absence of amyloid. To test this, IBA1+ myeloid cell numbers were determined. There was a significant difference in the numbers of IBA1+DAPI+ cells in WT mice of different strains (**Fig 6**). WT mice from CAST and WSB mice showed significantly fewer IBA1+ cells compared to B6 (Bonferroni’s multiple comparison test vs B6: Male WSB p ≤ 0.0001 and CAST p ≤ 0.01; Female WSB p ≤ 0.01 and CAST p ≤ 0.05). This supports the transcriptional profiling data (**Fig 5**). As expected, there was a significant sex and region-specific increase in IBA1+ cells in mice carrying the *APP/PS1* transgenes compared to their WT counterparts (**Fig 6, S3 Table**).

**Fig 7:**
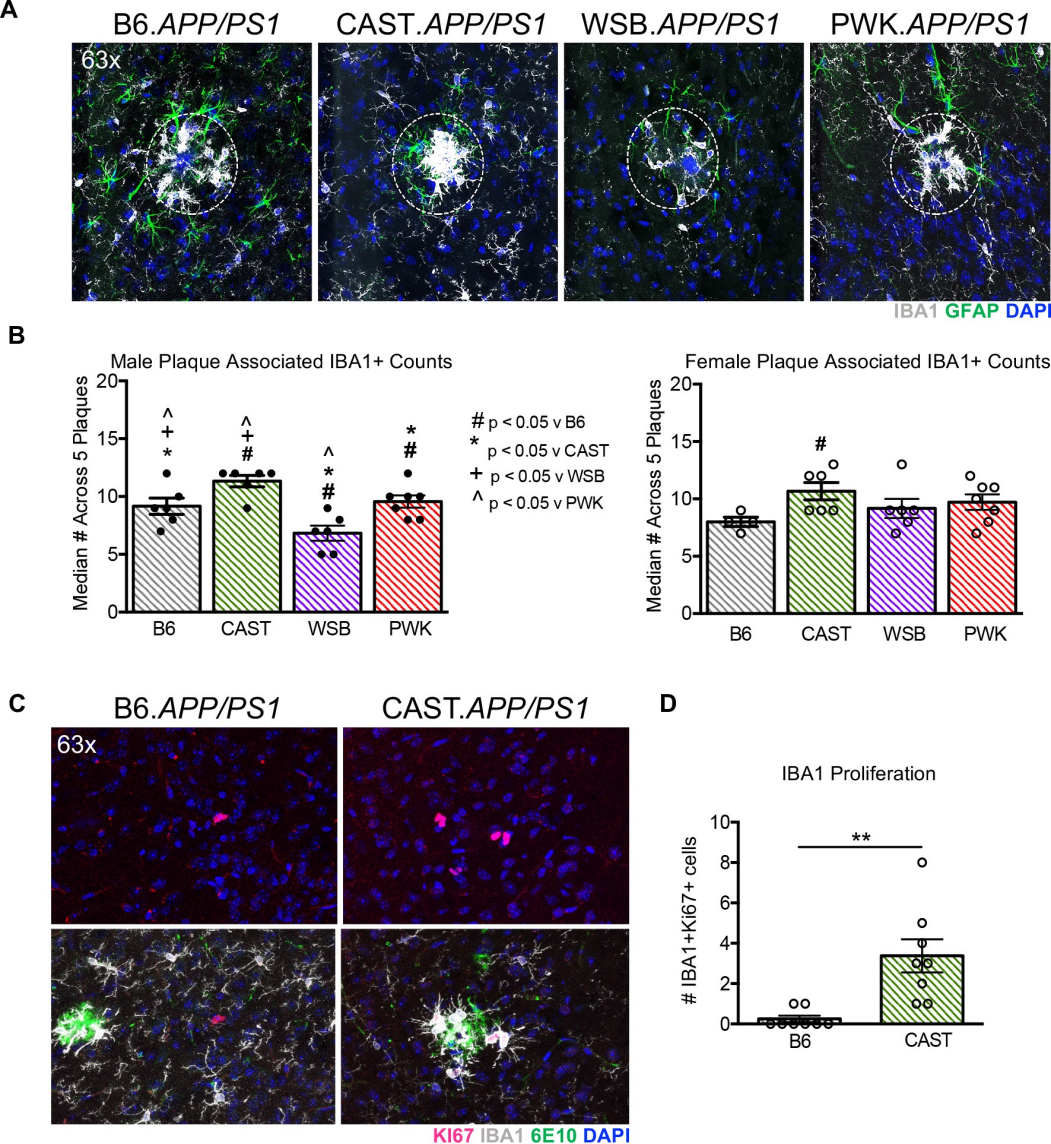
Myeloid cell response to amyloid is different across strains. (A) Representative 63x images of glial cells (IBA1+ myeloid cells and GFAP+ astrocytes) in *APP/PS1* (lower) for each strain. Scale bar represents 50 microns. Dashed circles identify relative size of plaques for IBA1+ cell counts. (B) IBA1+ myeloid cells were counted across five plaques of similar relative size for 6 *APP/PS1* per sex per strain (except for B6.*APP/PS1* females where only 4 samples were counted). There was a significant strain- and sex-dependent effect on myeloid cell numbers in *APP/PS1* mice as revealed through multiple comparisons. Symbols indicate significant differences (p ≤ 0.05) when comparing a single strain to B6 (#), CAST (*), WSB (+) and PWK (^). (C) Representative 20x images of Ki67+ staining in female B6.*APP/PS1* and CAST.*APP/PS1*. While there is positive Ki67 staining in B6.*APP/PS1*, the staining does not co-localize with IBA1 or 6e10. In CAST.*APP/PS1*, Ki67 staining is prominently co-localized with IBA1+ cells and 6e10. (D) The number of IBA1+Ki67+ was counted in female CAST.*APP/PS1* mice and was significantly increased (p ≤ 0.01) compared to female B6.*APP/PS1*.

Transcriptional profiling also predicted that plaque-mediated myeloid cell responses would differ between strains. To assess this, the numbers of myeloid cells surrounding plaques were determined for each *APP/PS1* strain. For each mouse, the numbers of IBA1+DAPI+ cells (myeloid) were determined around five plaques of similar relative size in 6 mice per strain (a total of 30 plaques/strain, **Fig 7A and 7B**). The median number of IBA1+ myeloid cells per section was averaged per animal and then compared across strains. For male animals, CAST.*APP/PS1* had the greatest number of plaque-associated IBA1+ cells, while WSB.*APP/PS1* had the least. For female animals, CAST.*APP/PS1* exhibited the greatest number of plaque-associated IBA1+ cells.

One myeloid cell response that has recently been highlighted as important is proliferative capacity, and there remains a debate regarding whether this is helpful or harmful in response to injury or in progression of neurodegenerative diseases [33, 34]. CAST.*APP/PS1* mice showed the greatest numbers of myeloid cells around plaques despite having the fewest numbers of myeloid cells in WT animals (**Fig 6**). To determine whether this could be due to myeloid cell proliferation, the proliferative marker KI-67 was used. KI-67+IBA1+ cells in CAST.*APP/PS1* mice were compared to B6.*APP/PS1* mice. There were significantly more plaque associated KI-67+IBA1+ cells observed in CAST.*APP/PS1* compared to B6.*APP/PS1* mice (t(14) = 3.73, p = 0.002) (**Fig 7D**). This suggests underlying differences in the proliferative capacity of myeloid cells between strains, and may be a factor in neuronal cell loss exhibited by CAST.*APP/PS1*.

## Discussion

The work presented here highlights the value and power of increased genetic diversity within mouse models in order to gain insight into the complex etiologies of human disease. Our work shows that in contrast to the B6.*APP/PS1* strain that has been widely used historically, CAST.*APP/PS1*, WSB.*APP/PS1* and PWK.*APP/PS1* represent models that provide a new lens to understanding central features of human AD including amyloid-induced neurodegeneration, neuroinflammation, cerebrovascular integrity and cerebral amyloid angiopathy. Overall, there were three major findings: (1) Female WSB.*APP/PS1* showed significant hippocampal and cortical neuronal cell loss, whole brain elevated levels of Aβ42, and cognitive impairment in a short-term memory task. This was accompanied by substantial vascular amyloid deposition in the form of cerebral amyloid angiopathy accompanied by vascular compromise. (2) CAST.*APP/PS1* showed hippocampal cell loss and females exhibited whole brain elevated levels of Aβ42. (3) Transcriptional profiling corroborated by neuropathology identified strain-dependent baseline differences in the expression of neuroinflammatory (primarily myeloid-related) genes and in the magnitude of their response to amyloid. Based on these findings, we predict that a major driver of the phenotype differences observed between strains (e.g. neuronal cell loss and CAA) is due to differences in neuroinflammation, particularly myeloid cells. A substantial part of the observed variation in myeloid cell-driven inflammation has been linked to genetic differences between human populations [35].

Wild-derived AD mouse models appear to show important strain- and sex-dependent differences in behavior and pathology that are similar to the human clinical population that show both sex-specific and ethnic differences in terms of prevalence and progression [36]. However, a major challenge of this study was to develop a functional battery that could be used across the strains as they exhibit formidable differences in wildness compared to classical laboratory strains. Wildness score is comprised of measurements of jumping, escape, struggle, squeaking and biting, and strains like B6 and D2 earn a score ranging between 0.21 and 0.66, while wild-derived strains range from 1.35 for CAST [26] to 2.5 or greater for WSB [27]. While it was important to include a range of functional assays, we anticipated there could be issues as traditional behavioral assays have been primarily optimized for typically behaving mice (e.g. young male B6) and likely would not be optimal for testing wild-derived strains. While B6.*APP/PS1* mice have previously been shown to exhibit deficits in assays such as Contextual Fear Conditioning as early as 6 months [37], we chose to avoid aversive tasks due to the inherent difficulty in handling wild-derived mice and stress caused to the animals with repeated handling. Instead, we chose tasks that utilized the animal’s natural exploratory drive such as y-maze tasks that assess spatial memory. While spatial memory deficits in B6 or B6/C3H mice carrying the APP/PS1 transgenes are typically over 12 months of age [38, 39], it would be expected that earlier impairment would be identified in a sensitized genetic context. To our knowledge, this is the first time that many of the tasks included in our battery were used to assess wild-derived mouse behavior, thus, all data is left intact (no outliers removed) and presented as individual data points. Unfortunately, in this study, not all WT strains were able to perform the novel spatial recognition task, which may have been due to the length of the memory delay chosen (30 minutes). This meant that short-term memory could not be assessed for some *APP/PS1* strains. Therefore, more extensive functional assays are still required at multiple ages to determine the utility of these strains for studies of cognitive impairment. Given our experiences in this study, it may be necessary to develop and validate strain-specific cognitive assays due to inherent differences in wildness and age-dependent cognitive abilities. This will be of particular importance in tasks that may require food restriction as these strains have vastly different metabolic rates.

In the age window tested, there were significant differences in plaque numbers and Aβ42 levels between strains. Taken alone, plaque counts would suggest less amyloid in the wild-derived *APP/PS1* mice in comparison with B6.*APP/PS1*. However, the size distribution of plaques varied across the strains, with B6.*APP/PS1* exhibiting many smaller proximal deposits that may correspond to initial seeding of Aβ. This is in contrast to plaques in the brains of wild-derived *APP/PS1* mice that were of moderate size. This could be indicative of a more advanced stage of amyloid deposition in 8 month wild-derived mice carrying *APP/PS1* in comparison with B6.*APP/PS1* of the same age; and/or, suggest the presence of different conformations of amyloid fibrils. Previous work has shown that identical peptide sequences are capable of forming into different conformations of amyloid fibrils, and that this difference can be detected by seeding efficiencies [40].

The development of cerebral amyloid angiopathy (CAA) in WSB.*APP/PS1* coupled with fibrin leakage (**Fig 4**) suggests compromised vascular integrity and/or deficits in amyloid clearance. It is possible that cerebrovascular damage (measured here by fibrin leakage) is downstream of amyloid. Conversely, an inherent weakness in cerebrovascular structures in WSB mice may dispose mice to CAA. While CAA has been reported many times before in AD models carrying APP mutations on a B6 background, typically it does not appear with complete banding until mice are over 14 months of age [41–43]. Furthermore, the severity of CAA and risk of associated microhemorrhage progresses with age. There is strong evidence to suggest that the earliest predictors of AD-susceptibility and onset are related to vascular and blood-brain-barrier integrity [44]. The presence of severe CAA and neurodegeneration in WSB.*APP/PS1* will allow mechanistic dissection of the relationship between CAA and neurodegeneration.

We found that strain is the greatest driver of gene expression variation in these mouse models, even more so than sex or *APP/PS1*. This is representative of the inclusion of millions of genetic differences created from wild-derived strains that have never before been explored in context of modeling AD. *Mus musculus*, also known as the house mouse, are characterized as being commensal animals, meaning that they live in close association with humans, and even though they are able to adapt to a wide-range of environments, are dependent on human shelter or activity for their survival [15]. Each distinct subspecies is from different geographical regions (CAST was trapped in Thailand, WSB was trapped in eastern shore Maryland, USA and PWK was trapped in the Czech Republic), and evolved separately to survive alongside humans in the face of similar region-specific pressures (exposure to pathogens or infection, climate, diet etc.). Therefore, some of the genetic differences between mouse substrains may correspond with genetic variants in different populations of humans. More likely however, the variations driving the phenotype differences will impact similar genes/pathways that are modified by genetic risk variants in the human population. In support of this, many of the genes in the module identified by WGCNA (**Fig 5D**) have previously been implicated in human AD–including sporadic AD–either through genetic association, gene expression studies or functional studies. Therefore, despite the presence of the *APP/PS1* transgene artificially driving amyloid accumulation in these strains–the responses appear to be directly relevant to human AD. This suggests that interventions tested in these new AD strains that target factors downstream of amyloid deposition but upstream of neurodegeneration would be expected to be clinically relevant to FAD and LOAD.

PWK.*APP/PS1* is particularly intriguing as transcriptional data suggest it is the greatest responder to amyloid at 8 months and appears to be a resilient strain (no neuronal cell loss detected). These data may be consistent with a slower progression/transition from amyloid deposition to neuronal cell dysfunction which could become apparent at older ages, or representative of a neuroprotective signature. Two additional and striking phenotypes may also be reflective of the substantial neuroinflammation in PWK.*APP/PS1* mice. First, during generation of the experimental cohort, PWK.*APP/PS1* had to be separated from WT littermates at 3 months of age due to increased aggression. Second, changes in activity in the piezoelectric chambers were observed in female PWK.*APP/PS1* (**S3 Fig**). These may be behavioral manifestations of increased neuroinflammation in response to amyloid. Agitation and circadian disruption are clinical symptoms that directly interfere with the ability of caregiving to occur in the home and have both been linked with neuroinflammation in humans [45, 46].

Transcriptional profiles in WT animals suggested that in comparison with B6 and PWK, CAST and WSB show lower baseline expression of the primarily myeloid-related genes in this module. Cell counts confirmed that there was ~50% reduction in the number of IBA1+ cells in both CAST and WSB. Natural inherent differences in neuroinflammation is important given the lack of studies into how genetic variation impacts glial cell development and homeostasis in the human population–an area that might be critical in predisposing to age-related diseases such as AD. Similarly, while there have been renewed efforts to characterize the immune systems in wild-caught mice [47], there still remains a dearth of knowledge regarding how genetic variation impacts myeloid cell and astrocyte function in inbred wild-derived strains. This may be starting to change as recent work by Christopher Glass and colleagues [48] analyzed macrophages from five inbred mouse strains, including PWK. As in our study, strain was the greatest driver of differences in gene expression in these macrophages.

Much of the foundation of mouse genetics has been focused on examination of a single genetic difference while holding all other genetic (i.e. strain background) and environmental influences constant. Somewhere along the way, limited resources and a wise desire for standardization restricted this examination to only one or two laboratory strains, despite efforts more than 4 decades ago to develop mouse resources such as the wild-derived strains and periodic suggestions of researchers past to expand beyond one strain [49–51]. Our study represents one of few studies to utilize natural genetic variation in mice to gain further insight in human AD. For the first time, we show neurodegeneration and mixed pathology in wild-derived strains carrying the *APP/PS1* transgenes. Interestingly, our data suggests B6 is a ‘resilient’ strain when considering neurodegeneration. This ‘resilience’ may be specifically driven by differences in myeloid-related neuroinflammation, and we predict that differences in myeloid cell biology in these new wild-derived AD mouse models will provide a much-needed platform for identification of novel genes/variants modifying susceptibility to neuronal cell loss.

One caveat of these new strains is that amyloid is driven by the *APP/PS1* transgenes. Transgenic overexpression of proteins can provide additional side effects and mutations in *APP* and *PSEN1* which may not be ideal to uncover the mechanisms of sporadic AD. A second caveat is that, despite neuronal cell loss, they appear to lack overt TAU pathology (S5 Fig). Therefore, further work is still needed to improve both the construct validity and the face validity of these new mouse models. Research is now focused on inducing sporadic AD in mice in the absence of transgenic overexpression of familial AD mutations. Mice differ from humans in both the APP and TAU proteins. The human APP protein is generally considered to be more amyloidogenic than the mouse and the ratios of the 3R and 4R isoforms of TAU are balanced in human adults, but not in adult mice. This may be a contributing factor to the apparent lack of TAU pathology in wild-derived *APP/PS1* strains. Multiple efforts, including our own, are improving the construct validity of AD mouse models by humanizing the *App* and *Mapt* loci and incorporating sporadic AD-relevant variants such as *APOE*^*E4*^ and *TREM2*^*R47H*^. The development of gene editing technologies such as CRISPR make these approaches feasible. However, our study and others [12] show it will be important that these efforts incorporate genetic diversity and natural genetic variation to improve both the face and predictive validity of these new mouse models.

## Materials and methods

### Ethics statement

All research was approved by the Institutional Animal Care and Use Committee (IACUC) at The Jackson Laboratory (approval number 12005). Animals were humanely euthanized with ketamine/xylazine mixture. Authors performed their work following guidelines established by the “The Eighth Edition of the Guide for the Care and Use of Laboratory Animals” and euthanasia using methods approved by the American Veterinary Medical Association.”

### Mouse strains and cohort generation

All mice were bred and housed in a 12/12 hours light/dark cycle on pine bedding and fed standard 6% LabDiet Chow. Experiments were performed on four strain genetic backgrounds: C57BL/6J, CAST/EiJ (JAX Stock #000928), WSB/EiJ (JR#001145), and PWK/PhJ (JR#003715). B6.Cg-Tg(APPswe, PSEN1dE9)85Dbo/Mmjax (JAX stock #005864), and referred to in this study as B6.*APP/PS1* mice, were obtained from the Mutant Mouse Resource and Research Center (MMRRC) at The Jackson Laboratory and backcrossed with the three different mouse strains for at least 6 generations to produce: CAST.*APP/PS1* (JAX Stock #25973), WSB.*APP/PS1* (JAX Stock #25970) and PWK.*APP/PS1* (JAX Stock #25971).

Generation of experimental cohorts consisted of 12 mice of each sex and genotype (*APP/PS1* carriers and littermate wild-type controls). Due to increased pup mortality in the wild-derived strains, once determined to be pregnant, female mice were removed from the mating and housed individually. During this time, they were also given BioServ Supreme Mini-treats (Chocolate #F05472 or Very Berry Flavor #F05711) in order to discourage pup cannibalism. Animals were initially group-housed during aging and then individually housed at the start of the behavioral testing battery. Due to severe aggression in PWK.*APP/PS1* mice, these mice were individually housed earlier at 3 months of age. There was also some cohort loss throughout the behavioral battery (i.e. seizure lethality mainly in B6.*APP/PS1*), so individual data points are shown for all assays where appropriate. Due to a substantial loss in the first cohort of male CAST.*APP/PS1* during the behavioral battery, a second cohort was generated and tested. Data were analyzed independently and combined if there were no significant differences in groups. For post-mortem characterization of AD phenotypes, brains from 6 males and 6 females at 8 months (±2 weeks) were assessed, with the exception of female B6.*APP/PS1*, where only an n of 4 survived to harvest.

### Behavioral and physiological assessment

A behavioral and physiological battery was designed in to order to test a wide range of AD-relevant phenotypes (**S1 Fig**). All testing was conducted by trained technicians in the Center for Biometric Analysis at The Jackson Laboratory. *APP/PS1* strains were scheduled as they became available and on average, the battery took about 6 weeks to complete. Testing always involved blinding and randomization of all littermates. An animal’s data was excluded from analysis if there was an indication from the technician that it should be (i.e. due to animal escaping prior to placement in assay, equipment failure, etc.). Summary tables report *n*’s used in analyses and individual data points are shown in all plots. Animals were then directly taken from the facility to the laboratory for harvesting.

***Piezoelectric floor monitoring*** (Signal Solutions, Lexington, KY) is a non-invasive, high throughput method of assessing sleep patterns through measurement of breath rate to classify animals as either awake or asleep [52, 53]. The piezoelectric pad is located on the cage floor and is sensitive to respiratory patterns. Pressure on these sensors converts analog input into an electric/digital signal. The system monitors activity by measuring the amplitude of the electrical signal, and comparing this to a signal threshold in order to classify the animal as either awake or asleep. Data was exported as either hourly percent activity or as hourly activity bout length over 5 days. Activity for the first day was excluded to allow for animal acclimation, and then averaged across sex and genotype.

***Stress induced Hyperthermia*** is an assay developed to detect typical stress responses determined by elevation in body temperature as the sympathetic nervous system is activated. Disruptions in this response can be indicative of metabolic dysfunction and/or an anxiety phenotype. The day prior to testing, mice were individually housed in standard cages. On testing day, animals were brought into the testing room and allowed to habituate for 60 minutes. Body temperature was taken at two time points separated by a 10-minute delay via a glycerol lubricated thermistor rectal probe (Braintree Scientific). In between readings, mice were placed back into the home cage.

***Open field*** is a measure of exploration and motor activity. Introduction of *APP/PS1* on the B6 background has been correlated with hyperactivity, thus, this is an important measure for the battery to ensure that strain-specific differences in other tasks cannot be accounted for by hyperactivity alone. The apparatus used for this test was a square chamber (~40 x 40 x 40 cm) fabricated from clear Plexiglas and illuminated at 400 lux. Data are recorded via a sensitive infrared photobeam system in 5-minute time bins for a 60-minute trial length.

***Spontaneous Alternation Y-maze task*** is a widely used task to assess spatial working memory, and relies on the animal’s natural exploratory behavior [54]. Sequence of entries into each arm of the Y-maze is tracked to assess if animals demonstrate intact working memory. The maze is a y-shaped arena constructed of Plexigas with equal arm lengths (~30 cm), arm lane width (~6 cm) and wall height (~15 cm). Special arm covers were fabricated to ensure that wild-derived mice could not escape and were used for all strains. A black curtain surrounded the perimeter of the maze in order to minimize additional room cues. A camera mounted above recorded mouse exploration and tracking software (Noldus Ethovision) allowed the export of multiple variables such as sequence of arm entries. Mice were allowed to habituate to the testing room for 60 minutes prior to testing. Activity was recorded over an 8-minute period. Each maze was wiped out with 70% ethanol between animals. A correct alternation represented when animals entered three different arms of the Y-maze without returning to a previously visited arm. The initial two arm entries were subtracted from the total to account for the placement of the animal in arm A at the start of the trial. Percent correct was determined by dividing the number of correct alternations by the adjusted total arm entries throughout the trial. This means for our assay, performance at chance is calculated at 22%. Typical performance of a young B6 male is approximately 50% [55].

***Spatial working memory y-maze task*** was conducted at least 2 weeks after spontaneous alternation in the same y-maze arena. This task consisted of two trials separated by a 30-minute delay period. During trial 1, the start and familiar arms were available for the animals to explore for 10-minutes and featured two distinct intramaze cues. Animals were then returned to their home cage for the 30-minute delay. In trial 2, all three arms were open for exploration and time spent in each of the arms over the 5-minute period was calculated. An animal was determined to have intact short term memory if it spent significantly more time in the novel arm in comparison with the start and familiar arms. Between trials and between animals the maze was cleaned with 70% ethanol. An additional exclusion criteria for this task was exclusion for failure to explore both arms during trial one (≤20% time percentage per arm).

***Body composition measurements*** were collected at the conclusion of the battery if a significant weight difference was detected. Animals were weighed and placed into a Plexiglas tube of 2.5 inches diameter and 8 inches in length. This tube is placed in a nuclear magnetic resonance device (EchoMRI, Houston, TX) that uses a 5 gauss magnet in order to pulse a magnetic field in a gradient across the animal to determine body composition consisting of lean muscle, fat and water. Each scan lasts approximately 1–3 minutes. Upon conclusion, animals are returned to their home cage and the tube is cleaned with 70% ethanol.

### Tissue harvesting, protein isolation, and sectioning

A lethal dose of ketamine/xylazine was administered to mice by intraperitoneal injection, in accordance to IACUC protocols. After transcardial perfusion with 1X PBS (Phosphate buffered saline) brains were removed. The left hemisphere was snap frozen for RNA/protein isolation, and the right hemisphere was fixed in 4% paraformaldehyde for sectioning. Protein was extracted with Trizol Reagent (Life Technologies cat#15596–018) following manufacturer's guidelines. Pellets were resuspended in a solution of 1:1 8M urea and 1% SDS.

For tissue sectioning, following 48 hours in 4% paraformaldehyde, half brains were kept at 4°C and placed in 10% sucrose for 24 hours. The tissue was then placed in 30% sucrose for an additional 24 hours, or until it sank. Brains were then embedded in optimal cutting temperature (OCT) compound, sectioned at 25μm and stored at −80°C until required.

### Immunofluorescence, Thioflavin S and X34 staining

Primary antibodies were applied to 1xPBS washed brain sections and incubated for two nights at 4°C. The following primary antibodies were used to characterize neuronal and glial cells phenotypes in the brain: rabbit polyclonal anti-NeuN (1:200, Cell signaling), chicken polyclonal anti-GFAP (1:200, Acris Antibodies), rabbit polyclonal anti-IBA1 (1:200, Wako), and mouse polyclonal anti-Ki-67 (1:200, eBioscience). Primary antibodies were diluted in PBT (1X PBS, 1% TritonX-100) containing 10% normal donkey serum. After primary incubation, sections were washed three times in PBT and incubated with their respective secondary antibody (donkey anti-chicken Alexa Fluor 633 or donkey anti-rabbit Alexa Fluor 488/594, 1:1000 dilution, Life Technologies) for 2 hours at room temperature. All sections were then counterstained with DAPI and mounted with Aqua PolyMount. For Thioflavin S staining, sections were incubated with 1% Thioflavin S (diluted in a 1:1 water: ethanol ratio) for eight minutes at room temperature, followed by three washes in 80% ethanol, 95% ethanol, and finally in dH2O, and mounted. For assessment of cerebral amyloid angiopathy, X34 (100 uM, Sigma Aldrich) occurred first. Slides were brought to room temperature, washed with 1XPBS for 5 minutes and then incubated with 500 ul of the X34 solution. Slides were then dipped in deionized water and incubated with 500 ul of 0.02M NaOH for 5 minutes. After an additional wash with 1XPBS, primary antibodies rabbit polyclonal anti-fibrin (1:200, Millipore) and goat polyclonal anti-IBA1 (1:300, Abcam) were diluted in PBT and applied to the slide. The remainder of the protocol overlaps with that described above, with the exception of DAPI staining. For assessment of tau, X34 steps occurred first, followed by the use of Mouse on Mouse Basic Kit (Vector Laboratories). Tissue was then incubated ON in primary antibody Mouse Phospho-Tau (Ser202, Thr205) monoclonal antibody (AT8, 1:250, Thermofisher), goat anti-IBA1 (same as above) and rabbit Anti-NEUN (Abcam, 1:500) diluted in 10% normal donkey serum and PBT. Secondaries used overlap with description above. To ensure AT8 staining worked, a positive control slide of a 13 month hTau (B6.Cg-*Mapt*^*tm1(EGFP)Klt*^ Tg(MAPT)8cPdav/J, JR# 005491) was stained alongside *APP/PS1* strains. Images of IHC were taken using either the Leica SP5 confocal microscope, Leica SP8 confocal microscope or the Zeiss Axio Imager Z2.

### Image quantification

Quantitative analyses of plaques, microglial cells, astrocytes and neurons were performed in WT and *APP/PS1* mice of each of the four strains. The number of plaques present in the entire cortical region from three central sections for each mouse was determined. To quantify the number of IBA1+ microglia and GFAP+ astrocytes, 12 images (20X, 1388 X 1040 microns) were taken for each brain (for cortex: 3 images/section for 3 sections– 9 images in total; for hippocampus: 1 image in CA1/section for 3 sections– 3 images in total) with a Zeiss Axio Imager fluorescent microscope, and cells were manually counted using the cell counter plugin from ImageJ (1.47d) software. For counting NEUN+/DAPI neurons in the parietal cortex, three images (20X, 447 X 335 microns) were randomly taken in similar areas for each brain from each mouse, images were stacked using ImageJ and cropped altogether to 274.13 X 225.75 microns (including only cortical layers II and III). For quantification of pyramidal neurons in the hippocampus, images of the CA1 region were taken at 20X (447 X 335 microns) and cropped to 225.75 X 129.00 microns. NEUN+/DAPI+ cells in the cortex and hippocampus images were manually counted with the cell counter plugin from ImageJ (1.47d) software. All image analyses were performed blind to the experimental conditions. For IBA1+ cells surrounding plaques, five plaques per brain were imaged (using 20x optical lens). Images were processed and cells counted using the cell counter plugin for ImageJ/FIJI. For each mouse, IBA1+ cells around each plaque from the three images were totaled and then averaged across mice.

### Western blotting and ELISA

Protein levels of human APP were assessed using western blotting. Briefly, DC assay (Bio-Rad) was used to determine protein concentration. Protein samples were heated to 95°C for 5 minutes and a total of 10 ng of protein was loaded onto a 12% TGX stain-free gel (Bio-Rad). Protein samples were transferred to a nitrocellulose membrane (Life Technologies) using an iBlot (Thermo Fischer Scientific) following manufacturers’ instructions. Blots were then incubated overnight with 6E10 antibody (1:2000, Covance/BioLegend) and 5% milk/PBS-tween at 4°C. After incubation with the appropriate secondary antibodies (Anti-Mouse IgG 1:30,000, Millipore) for 2hrs at room temperature, ECL detection reagents (GE Healthcare) were used to develop the chemiluminescence signal. Blots were further probed with anti-GAPDH (1:1000, Millipore) after treatment with 0.1% sodium azide and incubation with secondary antibody (Anti-mouse IgG 1:30,000, Millipore) for 2 hours at room temperature, washed, and detected. Quantification of blots was determined using Fiji ImageJ software.

Human amyloid β-42 (Aβ42) levels were determined using the ELISA detection kit from Life Technologies (cat#KHB3442) following the manufacturer’s instructions. To ensure that urea and SDS levels in the protein samples were compatible with the ELISA kit (see protein isolation), protein samples from 8 months old mouse brains were diluted 1:50 in standard diluent buffer. Samples were then compared to a standard curve and Aβ42 concentrations were established against the samples’ protein concentrations following manufacturers recommendations.

### Transcriptional profiling

Left brain hemispheres (n = 93) were snap frozen at harvest and then samples corresponding to mice for pathological assessment were sent to The Jackson Laboratory Genome Technologies core for further processing. RNA was isolated from tissue using the MagMAX mirVana Total RNA Isolation Kit (ThermoFisher) and the KingFisher Flex purification system (ThermoFisher). Tissues were lysed and homogenized in TRIzol Reagent (ThermoFisher). After the addition of chloroform, the RNA-containing aqueous layer was removed for RNA isolation according to the manufacturer’s protocol, beginning with the RNA bead binding step. RNA concentration and quality were assessed using the Nanodrop 2000 spectrophotometer (Thermo Scientific) and the RNA Total RNA Nano assay (Agilent Technologies).

Libraries were prepared by the Genome Technologies core facility at The Jackson Laboratory using the KAPA mRNA HyperPrep Kit (KAPA Biosystems), according to the manufacturer’s instructions. Briefly, the protocol entails isolation of polyA containing mRNA using oligo-dT magnetic beads, RNA fragmentation, first and second strand cDNA synthesis, ligation of Illumina-specific adapters containing a unique barcode sequence for each library, and PCR amplification. Libraries were checked for quality and concentration using the D5000 assay on the TapeStation (Agilent Technologies) and quantitative PCR (KAPA Biosystems), according to the manufacturers’ instructions. Libraries were pooled and sequenced by the Genome Technologies core facility at The Jackson Laboratory, 100 bp paired-end on the HiSeq 4000 (Illumina) using HiSeq 3000/4000 SBS Kit reagents (Illumina) at an average sequencing depth of ~100 million reads per sample (S1 Fig). 2x100 base length paired end reads were quality trimmed and filtered using Trimmomatic tool [56] and reads passing the quality filtering were mapped to the mouse mm10 reference genome using ‘STAR’ aligner [57]. Custom genomes were generated for CAST, PWK and WSB strains by incorporating REL-1505 variants into the reference genome [58]. RSEM software package [59] was used to estimate expression levels for all genes in Transcripts Per Million (TPM) unit based on Ensembl Release 84 transcriptome. HTSeq Python package was used to calculate raw read counts per transcript.

Genes that have an HTSeq estimated raw read count of less than 10 in more than 90% of the samples were considered noise and excluded from the analysis. Downstream analyses were performed on 17408 genes that passed the read count threshold. We applied variance stabilization transformation (vst) on raw read counts using DESeq2 R package. Principal Component Analysis (PCA) was applied on vst transformed read counts to identify clusters of samples and any potential outliers.

We then applied Weighted Gene Co-expression Network Analysis (WGCNA) algorithm [60] to identify co-expressed gene modules in our dataset. We extracted module’s eigengenes, which are equivalent to the first principal component, to represent the overall expression profiles of the modules. GO pathway enrichment analysis was performed using Homer tool [61].

### Statistical analysis

For behavioral analyses, groups of 12 mice per sex per strain per genotype allowed us to detect effects of greater than 1.15 standard errors at 80% power. Due to some premature death, not all groups contained 12 mice but sample sizes are stated clearly in the associated tables and all graphs show individual data points. For neuropathology and biochemistry, six biological replicates were assessed for all groups with the exception of B6 female APP/PS1 (n = 4) and PWK female WT (n = 5). Individual data points are shown on all graphs and associated tables include sample sizes. For data within strain and sex were analyzed as non-parametric using Mann-Whitney Rank Sum Test to account for departure for normality or using unpaired t-tests. One-way multifactorial analysis variance (ANOVA) followed by Bonferroni post-hoc tests for multiple comparisons were utilized for across strain differences. All statistical tests are labeled within data tables. Data were analyzed using GraphPad Prism software. *P* values are provided as stated by GraphPad and significance was determined with *P* values less than 0.05. When significance values were more than two decimal values, *P* values were presented as follows: p ≤ 0.01 (**),p ≤ 0.001 (***) or p ≤ 0.0001 (****).

### Data and mouse strain availability

These new strains are available through The Jackson Laboratory and all associated data is being made available through GEO and Accelerating Medicines Partnership-Alzheimer’s disease (AMP-AD) knowledge portal.

## Supporting information

S1 Fig Study design and key for graphs. (A) Timeline for behavioral testing. Animals were aged to 6 months and then were placed into the piezoelectric floor monitoring assay for 5 days. The following week animals were tested on stress-induced hyperthermia and open field. There was at least two weeks between Spontaneous alternation and Novel Spatial Recognition. Overall, on average, the testing battery took 6 weeks to perform.
(B) Key for all data presentations. Background strain color (B6: Gray; CAST: Green; WSB: Purple; PWK: Red) are based on the designated founder strain colors used with the Collaborative Cross and Diversity Outbred lines.
(C) Representative genotyping gel showing presence of *APP*^*swe*^ and *PSEN1*^*de9*^ transgenes in the new wild-derived *APP/PS1* and B6.*APP/PS1* strains. Sample order for each strain are: *APP/PS1* male, *APP/PS1* female, WT male, WT female. Two protocols can be used to identify the presence of the *APP*^swe^ mutation and the human *PSEN1*. For *APP*^swe^, control band size is 750 bp and transgene is 400 bp. For *PSEN1*, control band size is 324 and transgene is 608. All mice in this study were hemizygous.
(TIF)

S2 Fig Metabolic differences across strains. (A) Animals were weighed at 6 months prior to piezoelectric floor monitoring. Significant differences in weight were observed in male CAST.*APP/PS1* (p ≤ 0.05) and WSB.*APP/PS1* (p ≤ 0.01). Significant differences in weight were also observed in female WSB.*APP/PS1* (p ≤ 0.01) and female PWK. *APP/PS1* (p ≤ 0.05).
(B) Due to substantial weight difference in WSB.*APP/PS1*, Echo-MRI whole body NMR was employed to assess body composition characteristics in comparison with B6.*APP/PS1*.
(C) The weight increase in female WSB.*APP/PS1* was accounted for by a significant increase in lean mass (p ≤ 0.01).
(D) Lower body temperature has been associated with human aging and AD [64] so we assessed baseline body temperature and stress response using stress induced hypothermia. While there was a significant strain effect in baseline body temperature differences, there were no observed genotype differences.
(E) Female B6.*APP/PS1* failed to demonstrate the expected 1°C body temperature after 10 minutes for the second time point (p ≤ 0.05).
(TIF)

S3 Fig Differences in strain in acute and chronic measures of activity. (A) Disturbances in activity patterns and sleep behavior have been identified in human patients and may be an early indication (or contributor) of disease pathology [65–67]. Mice were placed in the piezoelectric floor monitoring chamber to assess activity over a five-day period. Overall, we found strain differences in relation to activity traces that may be reflective of previously reported differences in circadian rhythm and entrainment to light [21]. Female percent hourly inactivity was averaged across animals and strains starting at midnight of the first day. Black traces in each plot are representative of WT inactivity and colored dashed lines are representative of *APP/PS1*. Gray shading represents the dark phase of the 12:12 hour animal housing room light cycle.
(B) Average percent hourly inactivity averaged across the 12 hour dark and light phase. Only PWK.*APP/PS1* females showed a significant difference in percent inactivity in comparison to WT (p ≤ 0.05), and were less active during the dark phase.
(C) A prominent sleep disturbance in AD patients is the emergence of shorter sleep bouts [68]. To determine if sleep bout differences were observed in different mouse strains, we examined the average length of time spent inactive per bout during light and dark phases. Female and male B6.*APP/PS1* mice had significantly shorter bouts during the dark phase. Surprisingly, irrespective of an overall change in activity, PWK.*APP/PS1* males showed significantly shorter inactivity bouts than WT males. However, comparison between WT male and female PWK animals revealed that males had significantly longer inactivity bouts than females, (Dark: t(10) = 3.91, p = 0.003; Light: t(10) = 3.24, p = 0.009).
(D) Female breath rate as measured by the piezoelectric floor was examined. Female CAST.*APP/PS1* mice showed a significant difference from WT counterparts (p ≤ 0.01).
(E) General exploratory and locomotor behavior was also assessed in an acute 60-minute (5-minute time bins) open field test. WT animals are represented with colored solid lines and *APP/PS1* are represented by dashed colored lines. Overall, wild-derived strains were significantly more active than their B6 counterparts. Previous studies had reported an increased activity (hyperactivity) in B6 mice carrying the *APP/PS1* transgenes compared to B6 mice [25], however we observed that this was significant in male, but not female B6.*APP/PS1* relative to sex-matched WT littermate controls. For the *APP/PS1* strains, the total distance traveled was significantly increased in both male and female WSB.*APP/PS1* compared to WT. Female CAST.*APP/PS1* also showed a significant increase in total distance traveled. There was no indication of anxiety related phenotypes or thigmotaxis as measured by time spent in the perimeter of the open field.
(F) Female total distance traveled summed across the entire 60 minute session. Female CAST.*APP/PS1* and WSB.*APP/PS1* were also significantly more active than WT counterparts (CAST.*APP/PS1* p ≤ 0.001; WSB.*APP/PS1* p ≤ 0.01).
(G) Novelty-induced rearing behavior is another measure typically reported and shown to be decreased in an anxiogenic environment [69]. Novelty-induced rearing was examined but no significant differences were found.
(TIF)

S4 Fig Assessment of working and short-term memory using Y-maze measures. (A) The Y-maze for spontaneous alternation is made of clear Plexiglas and is devoid of extra-maze cues. For these studies, a specially formulated cover was added to the Y-maze.
(B-C) There were no significant genotype differences observed in percent alternation or total maze entries for male or female mice. Dashed line on Spontaneous Alternation graphs corresponds to chance performance (22%) as re-entry into previous arm counts as an error. See S4 Table for associated statistical analyses in supporting information.
(D) The Y-maze for novel spatial recognition consists of three distinct intra-maze cues positioned at the end of each arm. During trial 1, the Novel arm was blocked and animals were allowed to explore the arena for 10 minutes. Mice were removed to the home cage for 30 minutes at which time the blockade was removed and animals were placed back in the maze and allowed to freely explore all maze arms.
(E-F) Percent time spent in the start and familiar arms was compared to percent time spent in the novel arms during the 5 minute trial 2. While all data is shown, caution should be used in interpretation of transgenic animals performance if the age-matched littermate WT did not demonstrate preference for the novel arm (For males, this is B6 and WSB WT. For females, this is B6 and CAST WT.). Overall, male and female WT and transgenic PWK demonstrated the expected preference for the novel arm indicative of intact short-term memory. Female WSB WT also demonstrated the expected preference for the novel arm, while this was not seen in the female transgenic WSB.*APP/PS1*. This may be indicative of cognitive impairment corresponding to neuronal loss. See S5 Table for associated statistical analyses in supporting information.
(TIF)

S5 Fig Assessment of early tau hyperphosphorylation pathology. (A-D) Representative 20x images for colocalization of IHC cortical staining in each of the strains with AT8 (a marker of early hyperphosphorylation of tau), IBA1(myeloid cells), X34(plaque) and NEUN (neurons). The final image for each strain is 63x corresponding to the white square in the 20x merge image. AT8 staining primarily corresponds to plaque across all of the transgenic strains and not neurons. There is some evidence of increased vascular staining in wild-derived *APP/PS1* and WT samples compared to B6.*APP/PS1* and WT samples. This suggests that the affinity for the AT8 antibody to bind non-specifically to vessels is increased in the wild-derived strains.
(E) is representative 20x images of a brain from a 13-month hTau exhibiting robust AT8 colocalization with NEUN stained at the same time as the other sections included in this figure.
(TIF)

S6 Fig Presence of cerebral amyloid Angiopathy. (A) Representative 20x images of ThioS+ vessels with scale bar representing 50 microns.
(B) Pie charts showing the number of brain samples that exhibited ThioS+ vessels for a combined 12 male and female *APP/PS1* mice per strain. Male and female samples were combined as no sex difference was observed.
(C) 63x images of ThioS+ vessels also depicted in (A). Silver staining in WSB.*APP/PS1* highlights the banding pattern associated with CAA.
(TIF)

S7 Fig Transcriptional Profiling Quality. (A) Quality control of RNA-seq samples was performed. The data shows that the sequencing depth for individual samples across the strains was equivalent.
(TIF)

S8 Fig Transcript expression of *App* and *PSEN1*. (A) Overall *App* expression is plotted across the strains. Transgenic *APP/PS1* express a chimeric mouse/human amyloid precursor protein (Mo/HuAPP695swe) and exhibit approximately a 2 fold elevation in *App* as has been previously reported with this model.
(B) Overall *PSEN1* expression is plotted across the strains. Transgenic *APP/PS1* express a mutant human presenilin 1 with a deletion of exon 9 (*PSEN1*^*de9*^), thus the human sequence was used to identify expression differences. There are no significant differences in expression across the transgenic strains.
(C) *PSEN1* is located on human chromosome 14. Human transcript is not present in WT animals. While matching human reads are present in transgenic *APP/PS1*, there is a clear deletion of exon 9 across all strains. This map was produced using the Integrative Genomics Viewer[70].
(TIF)

S9 Fig Weighted gene co-expression analysis identified modules. (A) Weighted gene co-expression analysis identified a number of modules, however, the ‘light yellow’ module showed the strongest association with the *APP/PS1* transgene (genotype-driven).
(TIF)

S1 Table Associated statistical analyses for NEUN+DAPI cell counts. (DOCX)

S2 Table Associated statistical analyses for plaque counts. (DOCX)

S3 Table Associated statistical analyses for IBA1+DAPI+ cell counts. (DOCX)

S4 Table Associated statistical analyses for the spontaneous alternation assay. (DOCX)

S5 Table Associated statistical analyses for the novel spatial recognition assay. (DOCX)

## References

[1] Brookmeyer R, Abdalla N, Kawas CH, Corrada MM. Forecasting the prevalence of preclinical and clinical Alzheimer's disease in the United States. Alzheimers Dement. 2017 10.1016/j.jalz.2017.10.009 . 29233480 PMC5803316

[2] Wan He DG, Paul Kowal. An Aging World In: Bureau USC, editor. International Population Reports: U.S. Government Publishing Office; 2016.

[3] International AsD. World Alzheimer Report 2018 The state of the art of dementia research: New frontiers. London: 2018.

[4] Cummings J. Lessons Learned from Alzheimer Disease: Clinical Trials with Negative Outcomes. Clin Transl Sci. 2018;11(2):147–52. 10.1111/cts.12491 28767185 PMC5866992

[5] Ryman DC, Acosta-Baena N, Aisen PS, Bird T, Danek A, Fox NC, et al Symptom onset in autosomal dominant Alzheimer disease: a systematic review and meta-analysis. Neurology. 2014;83(3):253–60. Epub 2014/06/15. 10.1212/WNL.0000000000000596 24928124 PMC4117367

[6] Bennett DA, Buchman AS, Boyle PA, Barnes LL, Wilson RS, Schneider JA. Religious Orders Study and Rush Memory and Aging Project. J Alzheimers Dis. 2018;64(s1):S161–S89. Epub 2018/06/06. 10.3233/JAD-179939 . 29865057 PMC6380522

[7] Marioni RE, Harris SE, Zhang Q, McRae AF, Hagenaars SP, Hill WD, et al GWAS on family history of Alzheimer's disease. Transl Psychiatry. 2018;8(1):99 10.1038/s41398-018-0150-6 29777097 PMC5959890

[8] Lehman EJ, Kulnane LS, Gao Y, Petriello MC, Pimpis KM, Younkin L, et al Genetic background regulates beta-amyloid precursor protein processing and beta-amyloid deposition in the mouse. Hum Mol Genet. 2003;12(22):2949–56. 10.1093/hmg/ddg322 . 14506131

[9] Ryman D, Gao Y, Lamb BT. Genetic loci modulating amyloid-beta levels in a mouse model of Alzheimer's disease. Neurobiol Aging. 2008;29(8):1190–8. 10.1016/j.neurobiolaging.2007.02.017 17400334 PMC3745768

[10] Jackson HM, Onos KD, Pepper KW, Graham LC, Akeson EC, Byers C, et al DBA/2J genetic background exacerbates spontaneous lethal seizures but lessens amyloid deposition in a mouse model of Alzheimer's disease. PLoS One. 2015;10(5):e0125897 10.1371/journal.pone.0125897 25933409 PMC4416920

[11] Sittig LJ, Carbonetto P, Engel KA, Krauss KS, Barrios-Camacho CM, Palmer AA. Genetic Background Limits Generalizability of Genotype-Phenotype Relationships. Neuron. 2016;91(6):1253–9. 10.1016/j.neuron.2016.08.013 27618673 PMC5033712

[12] Neuner SM, Heuer SE, Huentelman MJ, O'Connell KMS, Kaczorowski CC. Harnessing Genetic Complexity to Enhance Translatability of Alzheimer's Disease Mouse Models: A Path toward Precision Medicine. Neuron. 2019;101(3):399–+. 10.1016/j.neuron.2018.11.040 WOS:000457856700014. 30595332 PMC6886697

[13] Morse HC, Cancer Research Institute., National Institute of Allergy and Infectious Diseases (U.S.). Origins of inbred mice: proceedings of a workshop, Bethesda, Maryland, February 14–16, 1978. New York: Academic Press; 1978. xvi, 719 p. p.

[14] Keane TM, Goodstadt L, Danecek P, White MA, Wong K, Yalcin B, et al Mouse genomic variation and its effect on phenotypes and gene regulation. Nature. 2011;477(7364):289–94. 10.1038/nature10413 21921910 PMC3276836

[15] Silver LM. Mouse genetics: concepts and applications. New York: Oxford University Press; 1995 xiii, 362 p. p.

[16] Deschepper CF, Olson JL, Otis M, Gallo-Payet N. Characterization of blood pressure and morphological traits in cardiovascular-related organs in 13 different inbred mouse strains. J Appl Physiol (1985). 2004;97(1):369–76. 10.1152/japplphysiol.00073.2004 . 15047670

[17] Lee KT, Karunakaran S, Ho MM, Clee SM. PWD/PhJ and WSB/EiJ mice are resistant to diet-induced obesity but have abnormal insulin secretion. Endocrinology. 2011;152(8):3005–17. 10.1210/en.2011-0060 . 21673102

[18] Ho MM, Hu X, Karunakaran S, Johnson JD, Clee SM. Altered pancreatic growth and insulin secretion in WSB/EiJ mice. PLoS One. 2014;9(2):e88352 10.1371/journal.pone.0088352 24505481 PMC3914989

[19] Kreznar JH, Keller MP, Traeger LL, Rabaglia ME, Schueler KL, Stapleton DS, et al Host Genotype and Gut Microbiome Modulate Insulin Secretion and Diet-Induced Metabolic Phenotypes. Cell Rep. 2017;18(7):1739–50. 10.1016/j.celrep.2017.01.062 28199845 PMC5325228

[20] Campbell JH, Foster CM, Vishnivetskaya T, Campbell AG, Yang ZK, Wymore A, et al Host genetic and environmental effects on mouse intestinal microbiota. ISME J. 2012;6(11):2033–44. 10.1038/ismej.2012.54 22695862 PMC3475380

[21] Jiang P, Striz M, Wisor JP, O'Hara BF. Behavioral and genetic dissection of a mouse model for advanced sleep phase syndrome. Sleep. 2011;34(1):39–48. 10.1093/sleep/34.1.39 21203370 PMC3001793

[22] Hu X, Crick SL, Bu G, Frieden C, Pappu RV, Lee JM. Amyloid seeds formed by cellular uptake, concentration, and aggregation of the amyloid-beta peptide. Proc Natl Acad Sci U S A. 2009;106(48):20324–9. 10.1073/pnas.0911281106 19910533 PMC2787156

[23] Jackson HM, Soto I, Graham LC, Carter GW, Howell GR. Clustering of transcriptional profiles identifies changes to insulin signaling as an early event in a mouse model of Alzheimer's disease. BMC Genomics. 2013;14:831 10.1186/1471-2164-14-831 24274089 PMC3907022

[24] Liu CC, Zhao N, Fu Y, Wang N, Linares C, Tsai CW, et al ApoE4 Accelerates Early Seeding of Amyloid Pathology. Neuron. 2017;96(5):1024–+. 10.1016/j.neuron.2017.11.013 WOS:000417336100011. 29216449 PMC5948105

[25] Rodgers SP, Born HA, Das P, Jankowsky JL. Transgenic APP expression during postnatal development causes persistent locomotor hyperactivity in the adult. Mol Neurodegener. 2012;7:28 10.1186/1750-1326-7-28 22709352 PMC3457908

[26] Wahlsten D, Metten P, Crabbe JC. A rating scale for wildness and ease of handling laboratory mice: results of 21 inbred strains tested in two laboratories. Genes Brain and Behavior. 2003;2(2):71–9. 10.1034/j.1601-183X.2003.00012.x WOS:000182147800002. 12884964

[27] Philip VM, Sokoloff G, Ackert-Bicknell CL, Striz M, Branstetter L, Beckmann MA, et al Genetic analysis in the Collaborative Cross breeding population. Genome Research. 2011;21(8):1223–38. 10.1101/gr.113886.110 WOS:000293335700003. 21734011 PMC3149490

[28] Sukoff Rizzo SJ, Anderson LC, Green TL, McGarr T, Wells G, Winter SS. Assessing Healthspan and Lifespan Measures in Aging Mice: Optimization of Testing Protocols, Replicability, and Rater Reliability. Curr Protoc Mouse Biol. 2018;8(2):e45 Epub 2018/06/21. 10.1002/cpmo.45 . 29924918

[29] Soto I, Grabowska WA, Onos KD, Graham LC, Jackson HM, Simeone SN, et al Meox2 haploinsufficiency increases neuronal cell loss in a mouse model of Alzheimer's disease. Neurobiol Aging. 2016;42:50–60. 10.1016/j.neurobiolaging.2016.02.025 27143421 PMC4878023

[30] Graham LC, Harder JM, Soto I, de Vries WN, John SWM, Howell GR. Chronic consumption of a western diet induces robust glial activation in aging mice and in a mouse model of Alzheimer's disease. Scientific reports. 2016;6:21568 Medline:26888450. 10.1038/srep21568 26888450 PMC4757836

[31] Arvanitakis Z, Capuano AW, Leurgans SE, Bennett DA, Schneider JA. Relation of cerebral vessel disease to Alzheimer's disease dementia and cognitive function in elderly people: a cross-sectional study. The Lancet Neurology. 2016;15(9):934–43. Medline:27312738. 10.1016/S1474-4422(16)30029-1 27312738 PMC4969105

[32] Scott TM, Bhadelia RA, Qiu WQ, Folstein MF, Rosenberg IH. Small Vessel Cerebrovascular Pathology Identified by Magnetic Resonance Imaging Is Prevalent in Alzheimer's Disease and Mild Cognitive Impairment: A Potential Target for Intervention. Journal of Alzheimer's disease: JAD. 2018;65(1):293–302. Medline:30040728. 10.3233/JAD-180366 30040728

[33] Gomez-Nicola D, Fransen NL, Suzzi S, Perry VH. Regulation of microglial proliferation during chronic neurodegeneration. The Journal of neuroscience: the official journal of the Society for Neuroscience. 2013;33(6):2481–93. Medline:23392676. 10.1523/JNEUROSCI.4440-12.2013 PMC6619184 23392676

[34] Fuger P, Hefendehl JK, Veeraraghavalu K, Wendeln A-C, Schlosser C, Obermuller U, et al Microglia turnover with aging and in an Alzheimer's model via long-term in vivo single-cell imaging. Nature neuroscience. 2017;20(10):1371–6. Medline:28846081. 10.1038/nn.4631 28846081

[35] Nedelec Y, Sanz J, Baharian G, Szpiech ZA, Pacis A, Dumaine A, et al Genetic Ancestry and Natural Selection Drive Population Differences in Immune Responses to Pathogens. Cell. 2016;167(3):657–69 e21. 10.1016/j.cell.2016.09.025 . 27768889

[36] As Association. 2017 Alzheimer's disease facts and figures. Alzheimer's & Dementia. 2017;13(4):325–73.

[37] Kilgore M, Miller CA, Fass DM, Hennig KM, Haggarty SJ, Sweatt JD, et al Inhibitors of Class 1 Histone Deacetylases Reverse Contextual Memory Deficits in a Mouse Model of Alzheimer's Disease. Neuropsychopharmacology. 2010;35(4):870–80. 10.1038/npp.2009.197 WOS:000274424900004. 20010553 PMC3055373

[38] Lalonde R, Kim HD, Maxwell JA, Fukuchi K. Exploratory activity and spatial learning in 12-month-old APP(695)SWE/co+PS1/DeltaE9 mice with amyloid plaques. Neurosci Lett. 2005;390(2):87–92. Epub 2005/09/20. 10.1016/j.neulet.2005.08.028 . 16169151

[39] Volianskis A, Kostner R, Molgaard M, Hass S, Jensen MS. Episodic memory deficits are not related to altered glutamatergic synaptic transmission and plasticity in the CA1 hippocampus of the APPswe/PS1deltaE9-deleted transgenic mice model of ss-amyloidosis. Neurobiol Aging. 2010;31(7):1173–87. Epub 2008/09/16. 10.1016/j.neurobiolaging.2008.08.005 . 18790549

[40] O'Nuallain B, Williams AD, Westermark P, Wetzel R. Seeding specificity in amyloid growth induced by heterologous fibrils. J Biol Chem. 2004;279(17):17490–9. 10.1074/jbc.M311300200 . 14752113

[41] Van Dorpe J, Smeijers L, Dewachter I, Nuyens D, Spittaels K, Van den Haute C, et al Prominent cerebral amyloid angiopathy in transgenic mice overexpressing the London mutant of human APP in neurons. Am J Pathol. 2000;157(4):1283–98. 10.1016/S0002-9440(10)64644-5 WOS:000089761200026. 11021833 PMC1850171

[42] Fryer JD, Taylor JW, DeMattos RB, Bales KR, Paul SM, Parsadanian M, et al Apolipoprotein E markedly facilitates age-dependent cerebral amyloid angiopathy and spontaneous hemorrhage in amyloid precursor protein Transgenic mice. Journal of Neuroscience. 2003;23(21):7889–96. WOS:000185001700021. 12944519 10.1523/JNEUROSCI.23-21-07889.2003 PMC6740607

[43] Shin HK, Jones PB, Garcia-Alloza M, Borrelli L, Greenberg SM, Bacskai BJ, et al Age-dependent cerebrovascular dysfunction in a transgenic mouse model of cerebral amyloid angiopathy. Brain. 2007;130:2310–9. 10.1093/brain/awm156 WOS:000250039300009. 17638859

[44] Iturria-Medina Y, Sotero RC, Toussaint PJ, Mateos-Perez JM, Evans AC, Alzheimer's Disease Neuroimaging I. Early role of vascular dysregulation on late-onset Alzheimer's disease based on multifactorial data-driven analysis. Nat Commun. 2016;7:11934 10.1038/ncomms11934 27327500 PMC4919512

[45] Musiek ES, Xiong DD, Holtzman DM. Sleep, circadian rhythms, and the pathogenesis of Alzheimer Disease. Exp Mol Med. 2015;47 ARTN e148 10.1038/emm.2014.121 WOS:000358592900005. 25766617 PMC4351409

[46] Ruthirakuhan M, Lanctot KL, Di Scipio M, Ahmed M, Herrmann N. Biomarkers of agitation and aggression in Alzheimer's disease: A systematic review. Alzheimer's & dementia: the journal of the Alzheimer's Association 2018. Medline:29940162. 10.1016/j.jalz.2018.04.013 29940162

[47] Abolins S, King EC, Lazarou L, Weldon L, Hughes L, Drescher P, et al The comparative immunology of wild and laboratory mice, Mus musculus domesticus. Nat Commun. 2017;8:14811 10.1038/ncomms14811 28466840 PMC5418598

[48] Glass DS, Riedel-Kruse IH. A Genetically Encoded Toolbox of Orthogonal Adhesins for Bacterial Self-Assembly. Biophys J. 2018;114(3):666a–a. WOS:000430563300322.

[49] Council NR. Mammalian models for research on aging: National Academies; 1981.

[50] Rivera J, Tessarollo L. Genetic background and the dilemma of translating mouse studies to humans. Immunity. 2008;28(1):1–4. 10.1016/j.immuni.2007.12.008 . 18199409

[51] Miller RA. Not Your Father's, or Mother's, Rodent: Moving Beyond B6. Neuron. 2016;91(6):1185–6. 10.1016/j.neuron.2016.09.009 27657444 PMC5495110

[52] Mang GM, Nicod J, Emmenegger Y, Donohue KD, O'Hara BF, Franken P. Evaluation of a piezoelectric system as an alternative to electroencephalogram/ electromyogram recordings in mouse sleep studies. Sleep. 2014;37(8):1383–92. 10.5665/sleep.3936 25083019 PMC4096208

[53] Yaghouby F, Donohue KD, O'Hara BF, Sunderam S. Noninvasive dissection of mouse sleep using a piezoelectric motion sensor. J Neurosci Methods. 2016;259:90–100. 10.1016/j.jneumeth.2015.11.004 26582569 PMC4715949

[54] Dudchenko PA. An overview of the tasks used to test working memory in rodents. Neurosci Biobehav Rev. 2004;28(7):699–709. 10.1016/j.neubiorev.2004.09.002 . 15555679

[55] Oades R, Taghzouti K, Simon H, Le Moal M. Dopamine-sensitive alternation and collateral behaviour in a Y-maze: effects of d-amphetamine and haloperidol. Psychopharmacology (Berl). 1985;85(1):123–8. Epub 1985/01/01. . 3920694 10.1007/BF00427335

[56] Bolger AM, Lohse M, Usadel B. Trimmomatic: a flexible trimmer for Illumina sequence data. Bioinformatics. 2014;30(15):2114–20. Epub 2014/04/04. 10.1093/bioinformatics/btu170 24695404 PMC4103590

[57] Dobin A, Davis CA, Schlesinger F, Drenkow J, Zaleski C, Jha S, et al STAR: ultrafast universal RNA-seq aligner. Bioinformatics. 2013;29(1):15–21. Epub 2012/10/30. 10.1093/bioinformatics/bts635 23104886 PMC3530905

[58] Choi KB, Vincent M.J., Churchill G.A. g2gtools: A Versatile Toolset for Custom Diploid Genome Creation and Coordinate Conversation. 2017 10.5281/zenodo.292952

[59] Li B, Dewey CN. RSEM: accurate transcript quantification from RNA-Seq data with or without a reference genome. BMC Bioinformatics. 2011;12:323 Epub 2011/08/06. 10.1186/1471-2105-12-323 21816040 PMC3163565

[60] Langfelder P, Horvath S. WGCNA: an R package for weighted correlation network analysis. BMC Bioinformatics. 2008;9:559 Epub 2008/12/31. 10.1186/1471-2105-9-559 19114008 PMC2631488

[61] Heinz S, Benner C, Spann N, Bertolino E, Lin YC, Laslo P, et al Simple combinations of lineage-determining transcription factors prime cis-regulatory elements required for macrophage and B cell identities. Mol Cell. 2010;38(4):576–89. Epub 2010/06/02. 10.1016/j.molcel.2010.05.004 20513432 PMC2898526

[62] Frazer KA, Eskin E, Kang HM, Bogue MA, Hinds DA, Beilharz EJ, et al A sequence-based variation map of 8.27 million SNPs in inbred mouse strains. Nature. 2007;448(7157):1050–3. 10.1038/nature06067 . 17660834

[63] Wade CM, Kulbokas EJ 3rd, Kirby AW, Zody MC, Mullikin JC, Lander ES, et al The mosaic structure of variation in the laboratory mouse genome. Nature. 2002;420(6915):574–8. Epub 2002/12/06. 10.1038/nature01252 . 12466852

[64] Tournissac M, Vandal M, Francois A, Planel E, Calon F. Old age potentiates cold-induced tau phosphorylation: linking thermoregulatory deficit with Alzheimer's disease. Neurobiol Aging. 2017;50:25–9. 10.1016/j.neurobiolaging.2016.09.024 . 27838492

[65] Ju YE, Lucey BP, Holtzman DM. Sleep and Alzheimer disease pathology—a bidirectional relationship. Nat Rev Neurol. 2014;10(2):115–9. 10.1038/nrneurol.2013.269 24366271 PMC3979317

[66] Mander BA, Winer JR, Jagust WJ, Walker MP. Sleep: A Novel Mechanistic Pathway, Biomarker, and Treatment Target in the Pathology of Alzheimer's Disease? Trends Neurosci. 2016;39(8):552–66. 10.1016/j.tins.2016.05.002 27325209 PMC4967375

[67] Kang DW, Lee CU, Lim HK. Role of Sleep Disturbance in the Trajectory of Alzheimer's Disease. Clin Psychopharmacol Neurosci. 2017;15(2):89–99. 10.9758/cpn.2017.15.2.89 28449556 PMC5426492

[68] Lim AS, Kowgier M, Yu L, Buchman AS, Bennett DA. Sleep Fragmentation and the Risk of Incident Alzheimer's Disease and Cognitive Decline in Older Persons. Sleep. 2013;36(7):1027–32. 10.5665/sleep.2802 23814339 PMC3669060

[69] Crawley JN. Unusual behavioral phenotypes of inbred mouse strains. Trends Neurosci. 1996;19(5):181–2; discussion 8–9. . 8723201 10.1016/s0166-2236(96)20021-9

[70] Robinson JT, Thorvaldsdottir H, Winckler W, Guttman M, Lander ES, Getz G, et al Integrative genomics viewer. Nature Biotechnology. 2011;29(1):24–6. 10.1038/nbt.1754 WOS:000286048900013. 21221095 PMC3346182

