# Supplementary figures and images for: Enhancing face validity of mouse models of Alzheimer’s disease with natural genetic variation
Source: PLoS Genet. 2019 May 31;15(5):e1008155. doi: 10.1371/journal.pgen.1008155 (PMC6576791; doi:10.1371/journal.pgen.1008155)

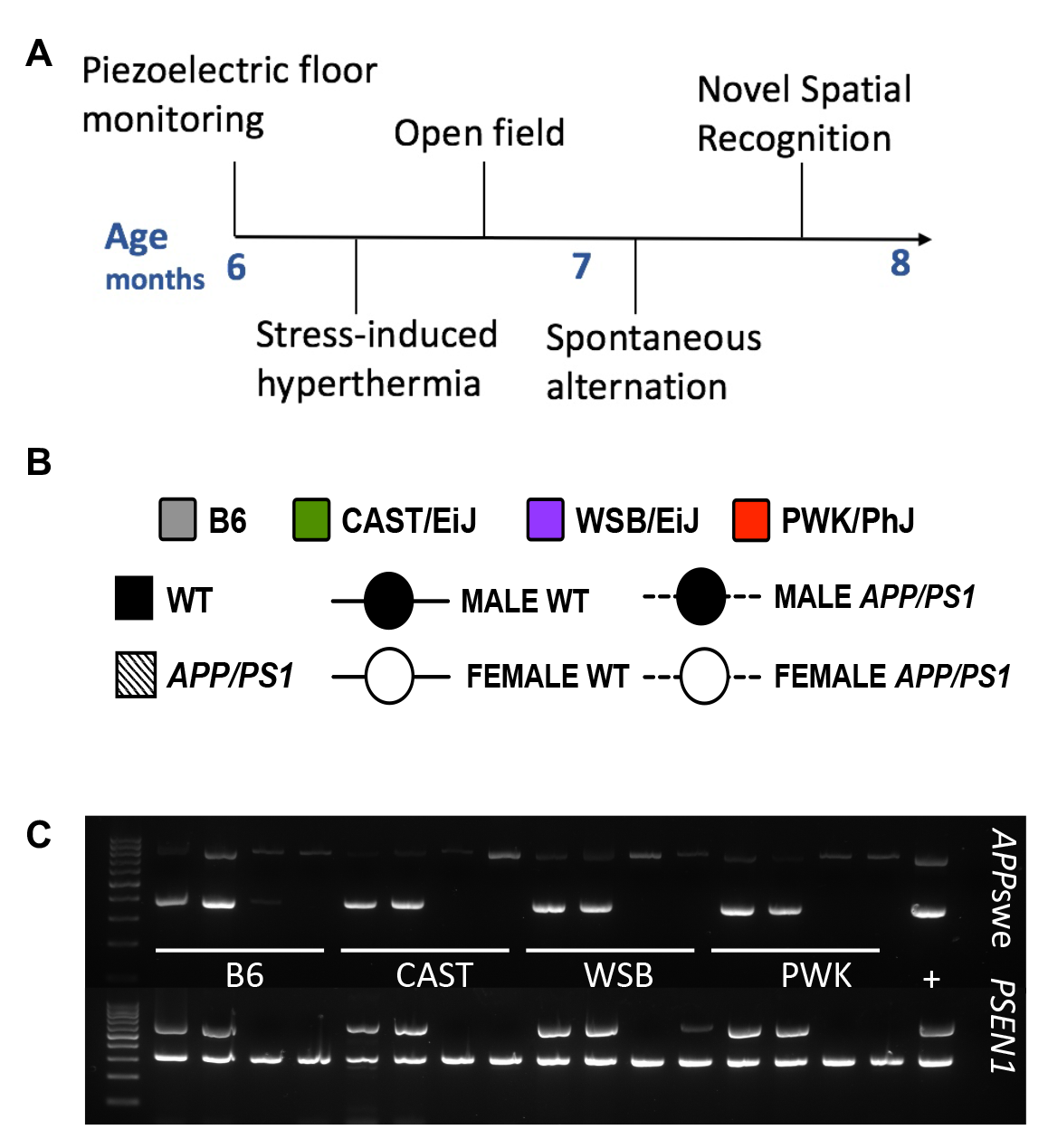

Supplement: S1 Fig — (A) Timeline for behavioral testing. Animals were aged to 6 months and then were placed into the piezoelectric floor monitoring assay for 5 days. The following week animals were tested on stress-induced hyperthermia and open field. There was at least two weeks between Spontaneous alternation and Novel Spatial Recognition. Overall, on average, the testing battery took 6 weeks to perform. (B) Key for all data presentations. Background strain color (B6: Gray; CAST: Green; WSB: Purple; PWK: Red) are based on the designated founder strain colors used with the Collaborative Cross and Diversity Outbred lines. (C) Representative genotyping gel showing presence of APPswe and PSEN1de9 transgenes in the new wild-derived APP/PS1 and B6.APP/PS1 strains. Sample order for each strain are: APP/PS1 male, APP/PS1 female, WT male, WT female. Two protocols can be used to identify the presence of the APPswe mutation and the human PSEN1. For APPswe, control band size is 750 bp and transgene is 400 bp. For PSEN1, control band size is 324 and transgene is 608. All mice in this study were hemizygous. (TIF) [file pgen.1008155.s001.tif]

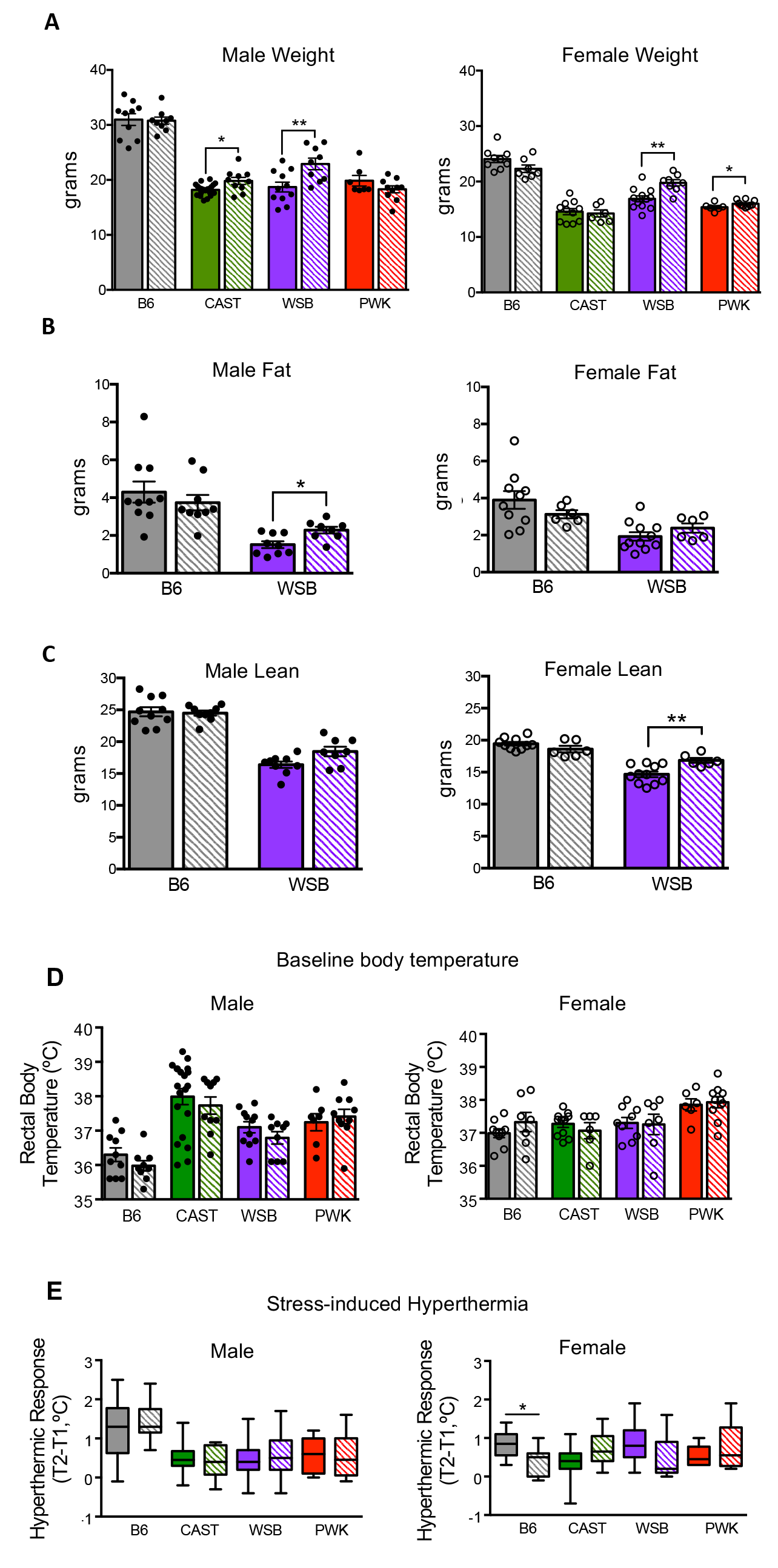

Supplement: S2 Fig — (A) Animals were weighed at 6 months prior to piezoelectric floor monitoring. Significant differences in weight were observed in male CAST.APP/PS1 (p ≤ 0.05) and WSB.APP/PS1 (p ≤ 0.01). Significant differences in weight were also observed in female WSB.APP/PS1 (p ≤ 0.01) and female PWK. APP/PS1 (p ≤ 0.05). (B) Due to substantial weight difference in WSB.APP/PS1, Echo-MRI whole body NMR was employed to assess body composition characteristics in comparison with B6.APP/PS1. (C) The weight increase in female WSB.APP/PS1 was accounted for by a significant increase in lean mass (p ≤ 0.01). (D) Lower body temperature has been associated with human aging and AD [64] so we assessed baseline body temperature and stress response using stress induced hypothermia. While there was a significant strain effect in baseline body temperature differences, there were no observed genotype differences. (E) Female B6.APP/PS1 failed to demonstrate the expected 1°C body temperature after 10 minutes for the second time point (p ≤ 0.05). (TIF) [file pgen.1008155.s002.tif]

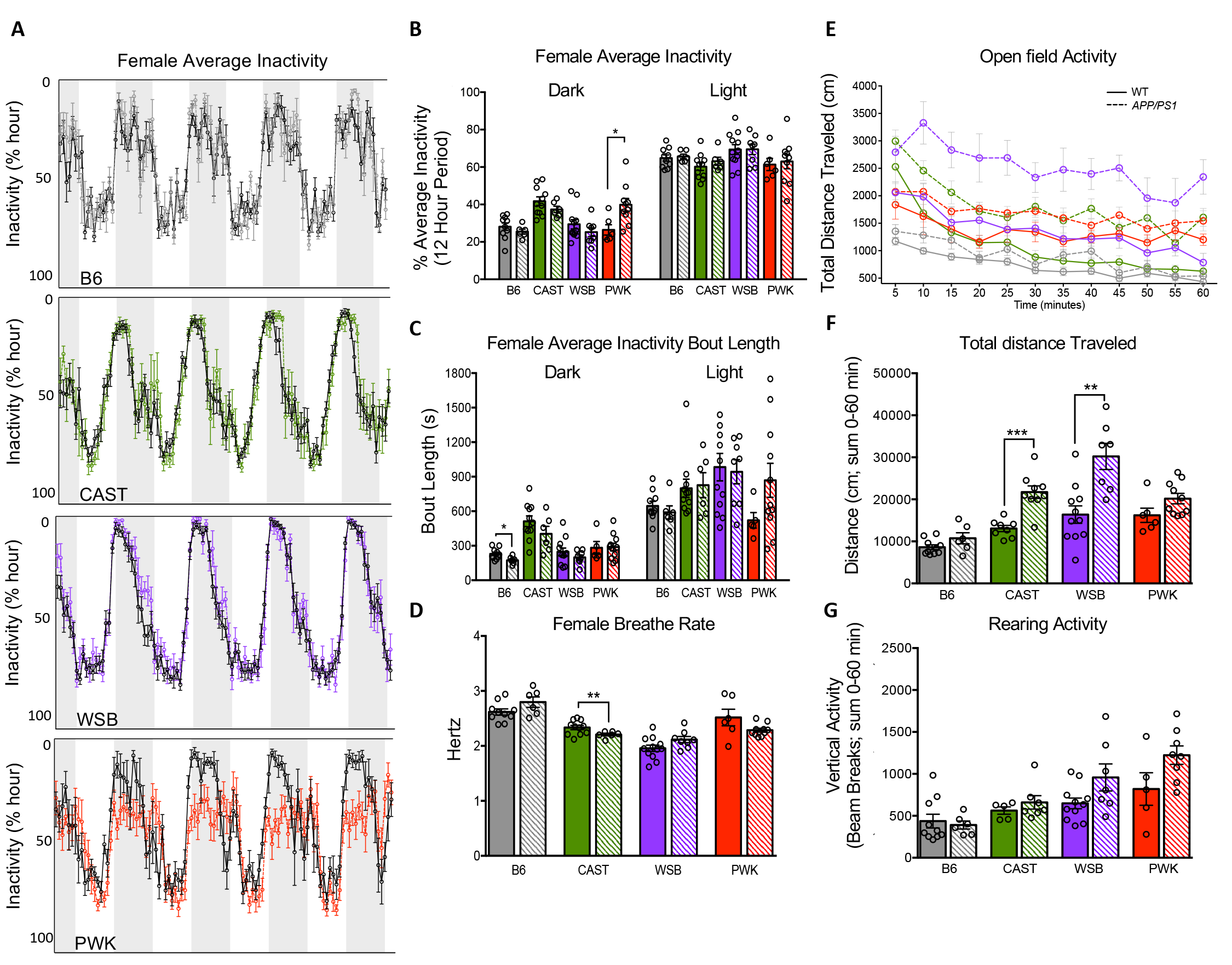

Supplement: S3 Fig — (A) Disturbances in activity patterns and sleep behavior have been identified in human patients and may be an early indication (or contributor) of disease pathology [65–67]. Mice were placed in the piezoelectric floor monitoring chamber to assess activity over a five-day period. Overall, we found strain differences in relation to activity traces that may be reflective of previously reported differences in circadian rhythm and entrainment to light [21]. Female percent hourly inactivity was averaged across animals and strains starting at midnight of the first day. Black traces in each plot are representative of WT inactivity and colored dashed lines are representative of APP/PS1. Gray shading represents the dark phase of the 12:12 hour animal housing room light cycle. (B) Average percent hourly inactivity averaged across the 12 hour dark and light phase. Only PWK.APP/PS1 females showed a significant difference in percent inactivity in comparison to WT (p ≤ 0.05), and were less active during the dark phase. (C) A prominent sleep disturbance in AD patients is the emergence of shorter sleep bouts [68]. To determine if sleep bout differences were observed in different mouse strains, we examined the average length of time spent inactive per bout during light and dark phases. Female and male B6.APP/PS1 mice had significantly shorter bouts during the dark phase. Surprisingly, irrespective of an overall change in activity, PWK.APP/PS1 males showed significantly shorter inactivity bouts than WT males. However, comparison between WT male and female PWK animals revealed that males had significantly longer inactivity bouts than females, (Dark: t(10) = 3.91, p = 0.003; Light: t(10) = 3.24, p = 0.009). (D) Female breath rate as measured by the piezoelectric floor was examined. Female CAST.APP/PS1 mice showed a significant difference from WT counterparts (p ≤ 0.01). (E) General exploratory and locomotor behavior was also assessed in an acute 60-minute (5-minute time bins) open field [file pgen.1008155.s003.tif]

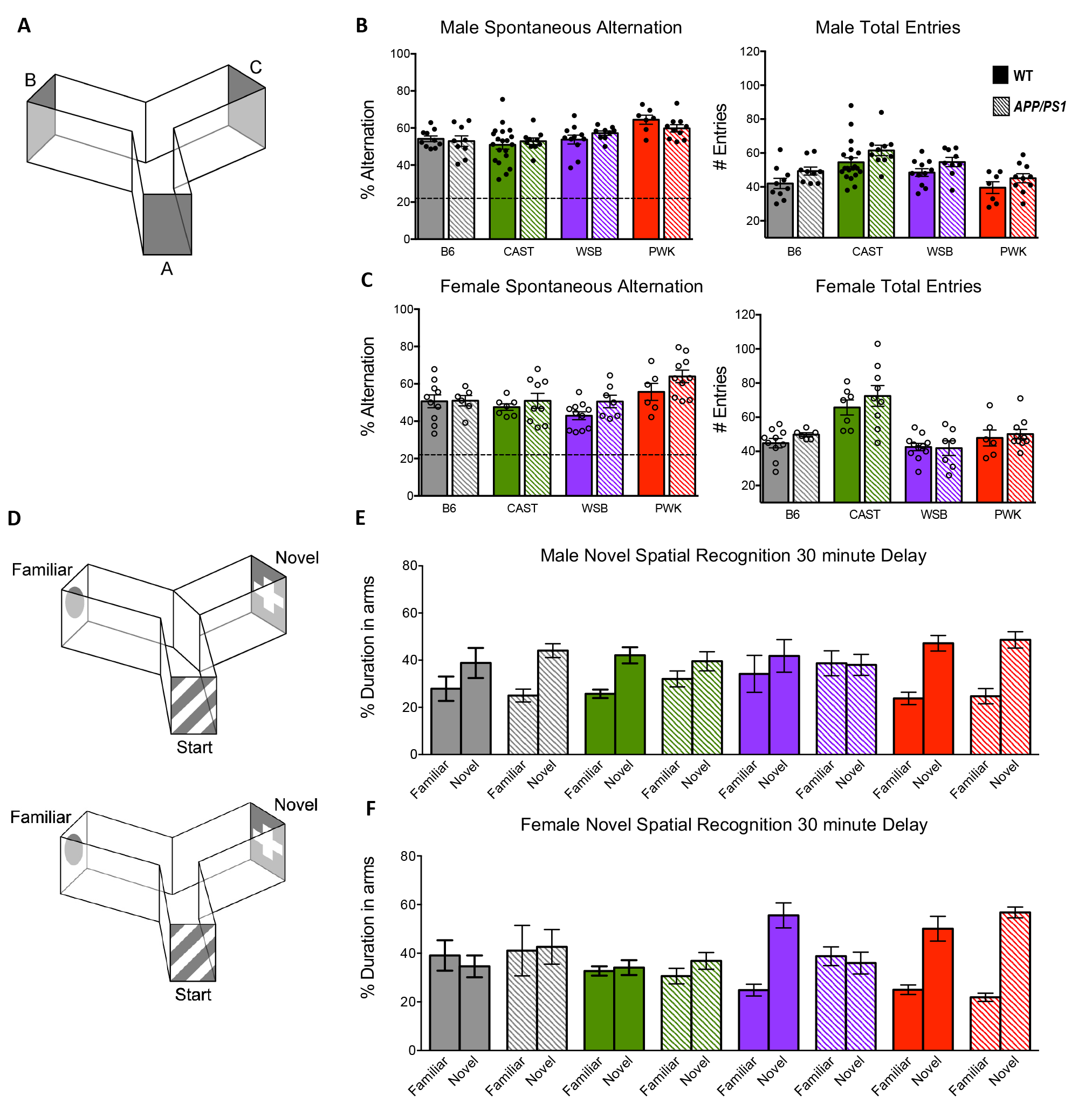

Supplement: S4 Fig — (A) The Y-maze for spontaneous alternation is made of clear Plexiglas and is devoid of extra-maze cues. For these studies, a specially formulated cover was added to the Y-maze. (B-C) There were no significant genotype differences observed in percent alternation or total maze entries for male or female mice. Dashed line on Spontaneous Alternation graphs corresponds to chance performance (22%) as re-entry into previous arm counts as an error. See S4 Table for associated statistical analyses in supporting information. (D) The Y-maze for novel spatial recognition consists of three distinct intra-maze cues positioned at the end of each arm. During trial 1, the Novel arm was blocked and animals were allowed to explore the arena for 10 minutes. Mice were removed to the home cage for 30 minutes at which time the blockade was removed and animals were placed back in the maze and allowed to freely explore all maze arms. (E-F) Percent time spent in the start and familiar arms was compared to percent time spent in the novel arms during the 5 minute trial 2. While all data is shown, caution should be used in interpretation of transgenic animals performance if the age-matched littermate WT did not demonstrate preference for the novel arm (For males, this is B6 and WSB WT. For females, this is B6 and CAST WT.). Overall, male and female WT and transgenic PWK demonstrated the expected preference for the novel arm indicative of intact short-term memory. Female WSB WT also demonstrated the expected preference for the novel arm, while this was not seen in the female transgenic WSB.APP/PS1. This may be indicative of cognitive impairment corresponding to neuronal loss. See S5 Table for associated statistical analyses in supporting information. (TIF) [file pgen.1008155.s004.tif]

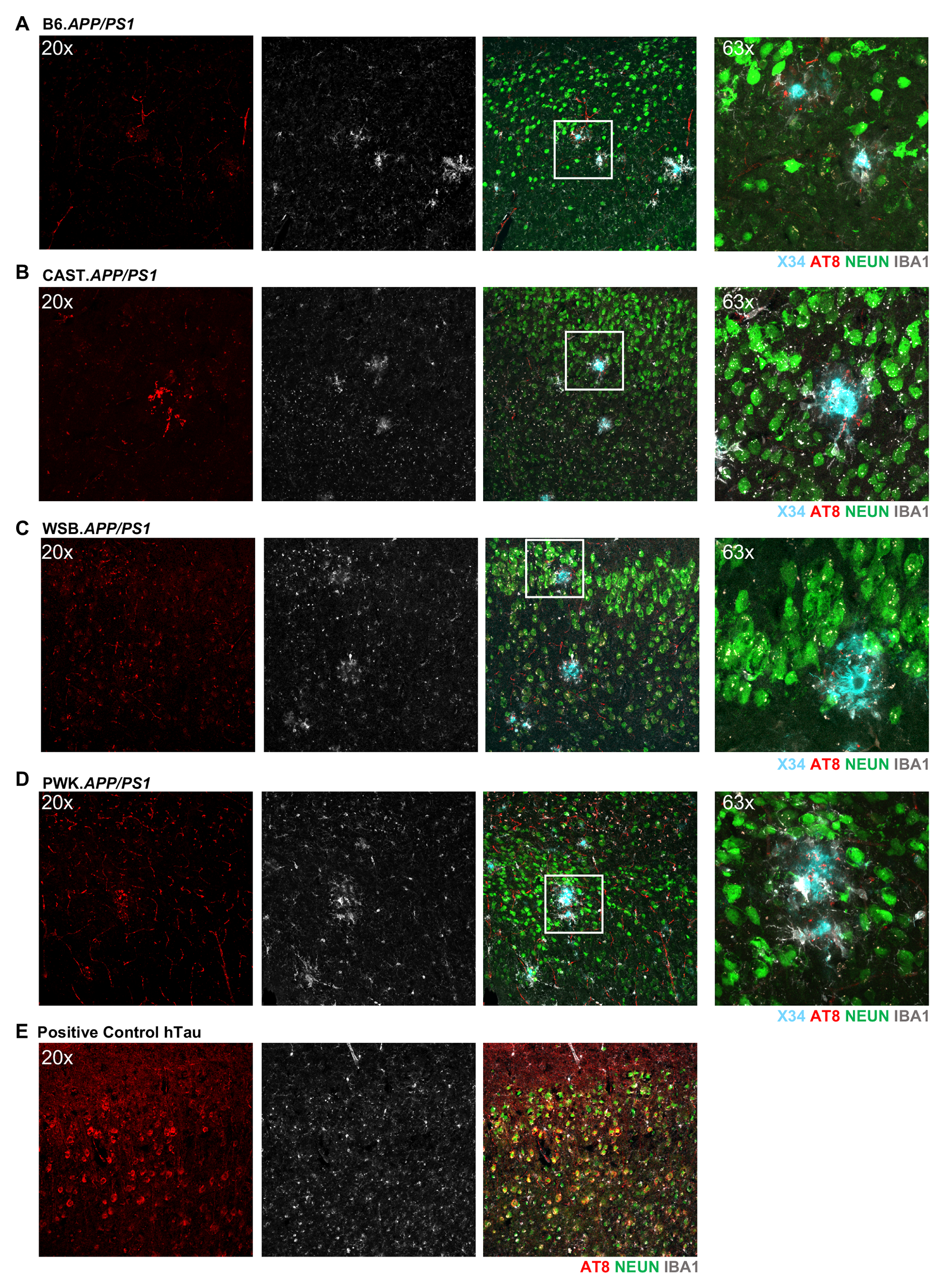

Supplement: S5 Fig — (A-D) Representative 20x images for colocalization of IHC cortical staining in each of the strains with AT8 (a marker of early hyperphosphorylation of tau), IBA1(myeloid cells), X34(plaque) and NEUN (neurons). The final image for each strain is 63x corresponding to the white square in the 20x merge image. AT8 staining primarily corresponds to plaque across all of the transgenic strains and not neurons. There is some evidence of increased vascular staining in wild-derived APP/PS1 and WT samples compared to B6.APP/PS1 and WT samples. This suggests that the affinity for the AT8 antibody to bind non-specifically to vessels is increased in the wild-derived strains. (E) is representative 20x images of a brain from a 13-month hTau exhibiting robust AT8 colocalization with NEUN stained at the same time as the other sections included in this figure. (TIF) [file pgen.1008155.s005.tif]

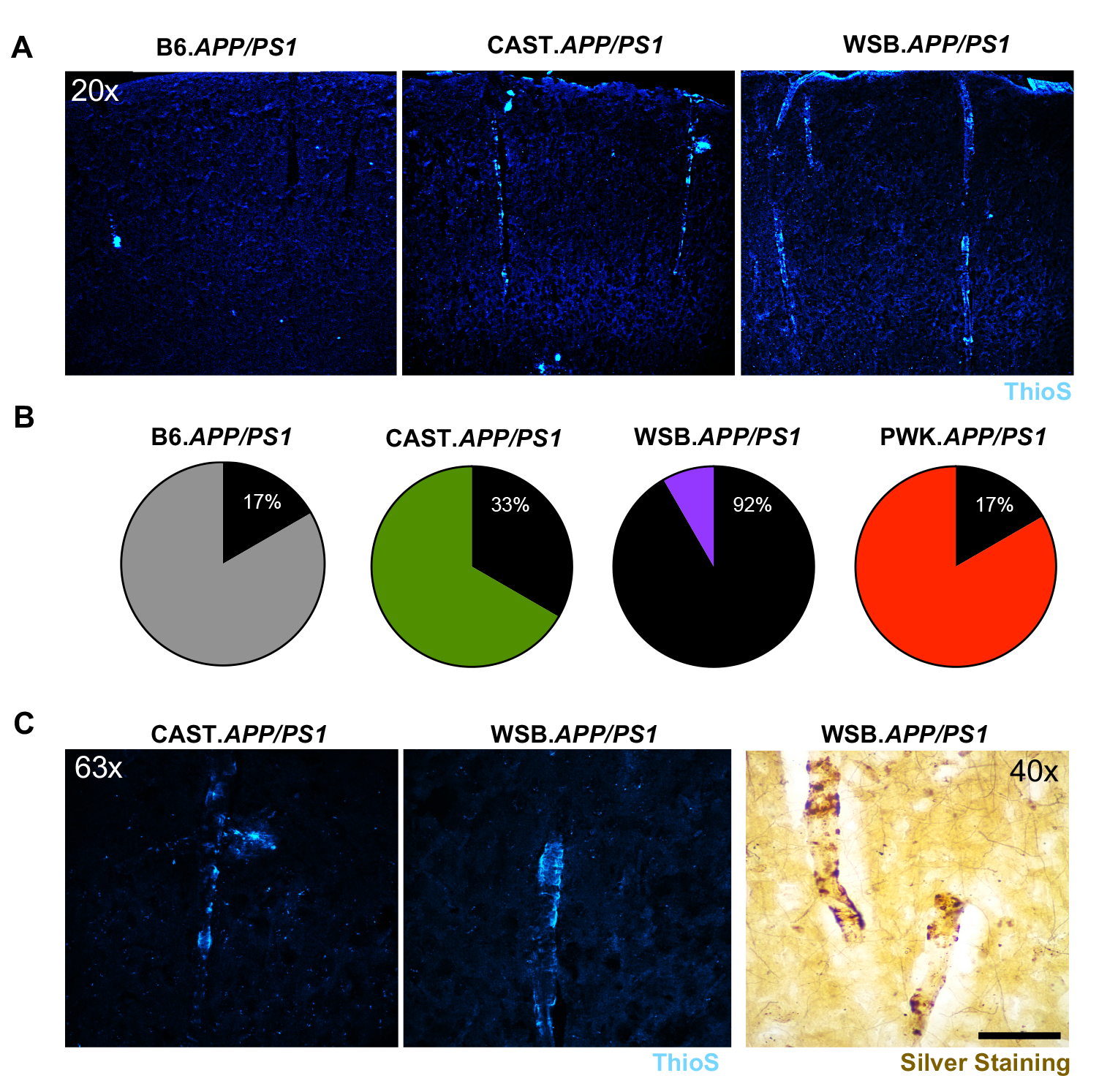

Supplement: S6 Fig — (A) Representative 20x images of ThioS+ vessels with scale bar representing 50 microns. (B) Pie charts showing the number of brain samples that exhibited ThioS+ vessels for a combined 12 male and female APP/PS1 mice per strain. Male and female samples were combined as no sex difference was observed. (C) 63x images of ThioS+ vessels also depicted in (A). Silver staining in WSB.APP/PS1 highlights the banding pattern associated with CAA. (TIF) [file pgen.1008155.s006.tif]

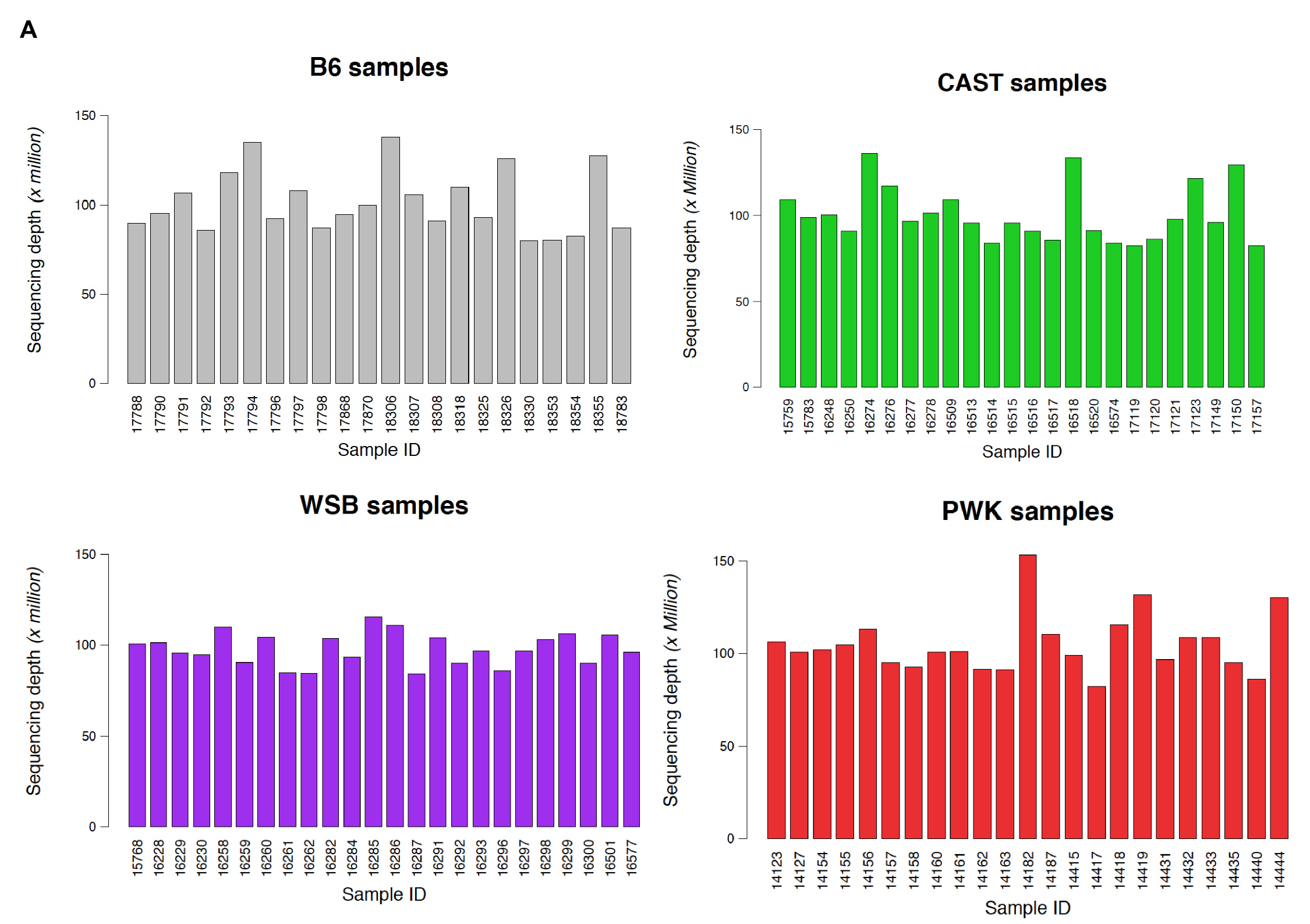

Supplement: S7 Fig — (A) Quality control of RNA-seq samples was performed. The data shows that the sequencing depth for individual samples across the strains was equivalent. (TIF) [file pgen.1008155.s007.tif]

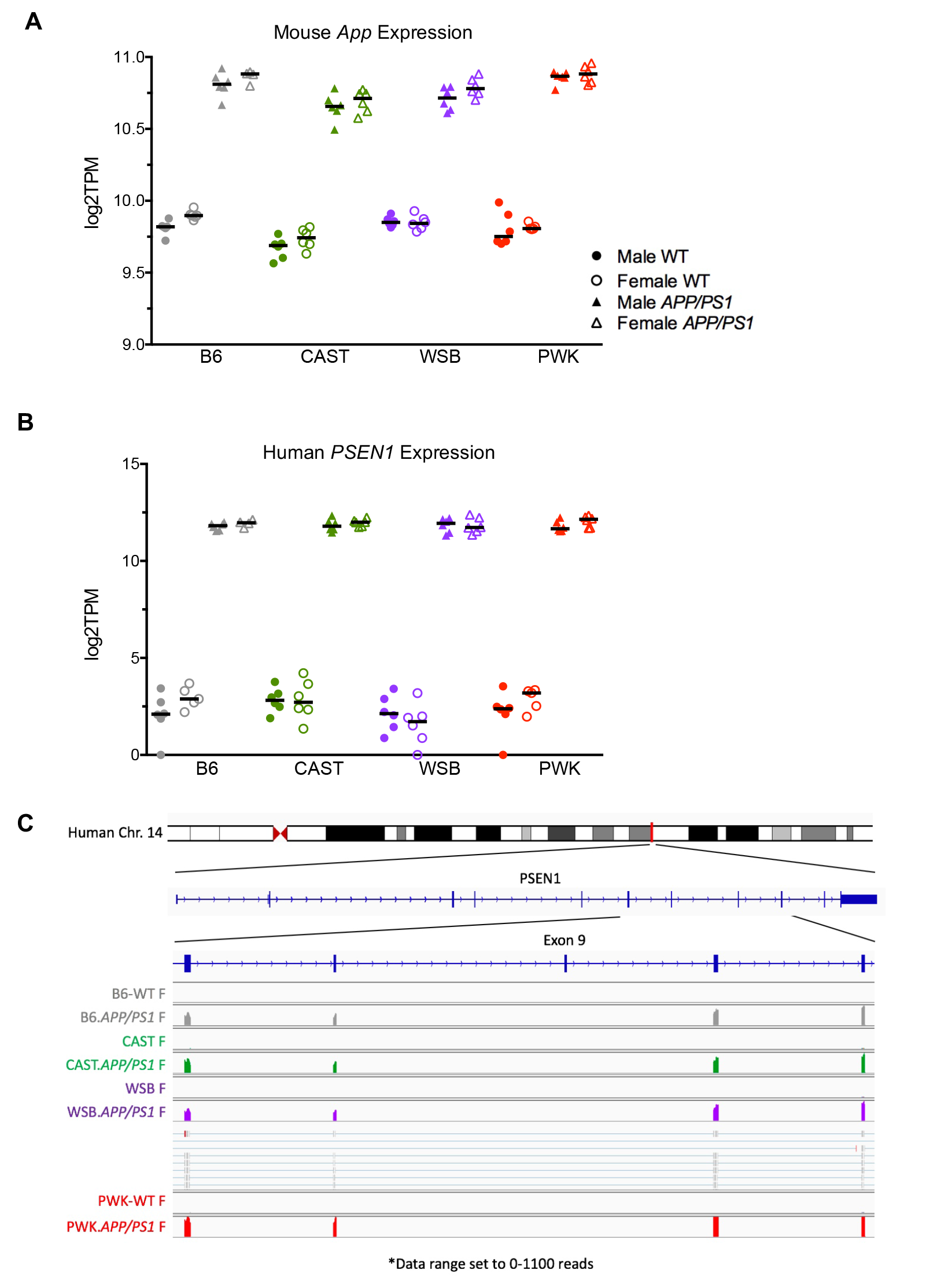

Supplement: S8 Fig — (A) Overall App expression is plotted across the strains. Transgenic APP/PS1 express a chimeric mouse/human amyloid precursor protein (Mo/HuAPP695swe) and exhibit approximately a 2 fold elevation in App as has been previously reported with this model. (B) Overall PSEN1 expression is plotted across the strains. Transgenic APP/PS1 express a mutant human presenilin 1 with a deletion of exon 9 (PSEN1de9), thus the human sequence was used to identify expression differences. There are no significant differences in expression across the transgenic strains. (C) PSEN1 is located on human chromosome 14. Human transcript is not present in WT animals. While matching human reads are present in transgenic APP/PS1, there is a clear deletion of exon 9 across all strains. This map was produced using the Integrative Genomics Viewer[70]. (TIF) [file pgen.1008155.s008.tif]

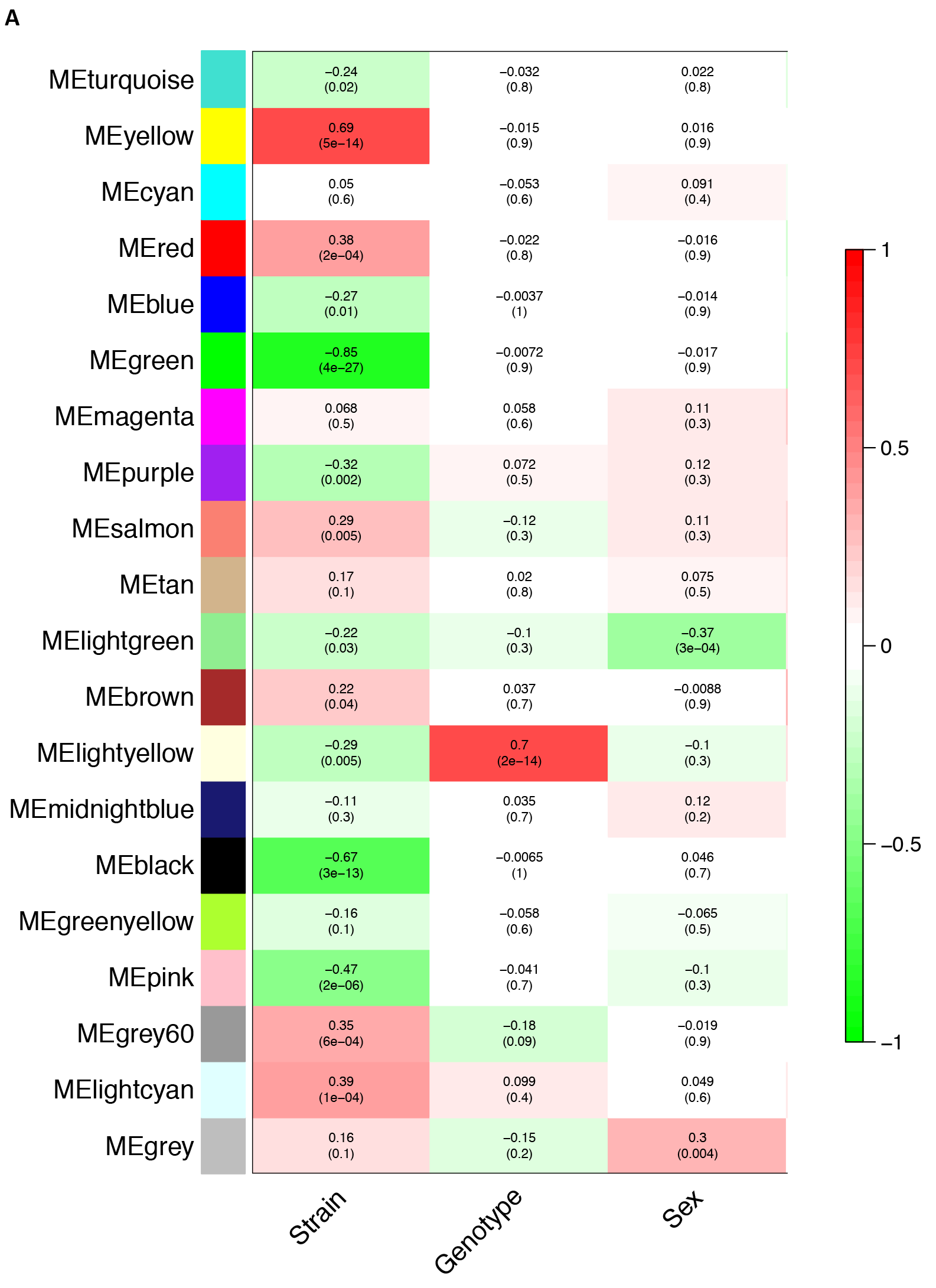

Supplement: S9 Fig — (A) Weighted gene co-expression analysis identified a number of modules, however, the ‘light yellow’ module showed the strongest association with the APP/PS1 transgene (genotype-driven). (TIF) [file pgen.1008155.s009.tif]
