# Supplementary material for: Enhancing face validity of mouse models of Alzheimer’s disease with natural genetic variation
Source: PLoS Genet. 2019 May 31;15(5):e1008155. doi: 10.1371/journal.pgen.1008155 (PMC6576791; doi:10.1371/journal.pgen.1008155)
Supplement: S5 Table — (DOCX) [file pgen.1008155.s014.docx]

**Table S5 – Novel Spatial Recognition**

* p < or equal to 0.05

** p < or equal to 0.01

*** p < or equal to 0.001

**** p < or equal to 0.0001
